# Organic Polyradicals as Redox Mediators: Effect of Intramolecular Radical Interactions on Their Efficiency

**DOI:** 10.1021/acsami.0c09386

**Published:** 2020-09-15

**Authors:** Elena Badetti, Vega Lloveras, Emanuele Amadio, Rosalia Di Lorenzo, Mara Olivares-Marín, Alvaro Y. Tesio, Songbai Zhang, Fangfang Pan, Kari Rissanen, Jaume Veciana, Dino Tonti, Jose Vidal-Gancedo, Cristiano Zonta, Giulia Licini

**Affiliations:** † Department of Chemical Sciences and CIRCC Padova Unit, University of Padova, Via Marzolo 1, 35131 Padova, Italy; ‡ Institut de Ciència de Materials de Barcelona (ICMAB-CSIC), Campus Universitari de Bellaterra, E-08193 Cerdanyola del Vallès, Spain; § Networking Research Center on Bioengineering, Biomaterials and Nanomedicine (CIBER-BBN), E-08193 Barcelona, Spain; ∥ Department of Mechanical, Energy and Materials Engineering, University Centre of Mérida, University of Extremadura, Avda. Santa Teresa de Jornet, 38, 06800 Mérida, Spain; ⊥ Centro de Investigación y Desarrollo en Materiales Avanzados y Almacenamiento de Energía de Jujuy (CIDMEJu), Centro de Desarrollo Tecnológico General Manuel Savio, Av. Martijena S/N, Palpalá Y 4612, Jujuy, Argentina; # Department of Chemistry, University of Jyvaskyla, P. O. Box 35, 40014 Jyväskylä, Finland

**Keywords:** redox mediators, spin−spin interactions, TEMPO, nitroxides, μ-oxo complexes, titanatranes

## Abstract

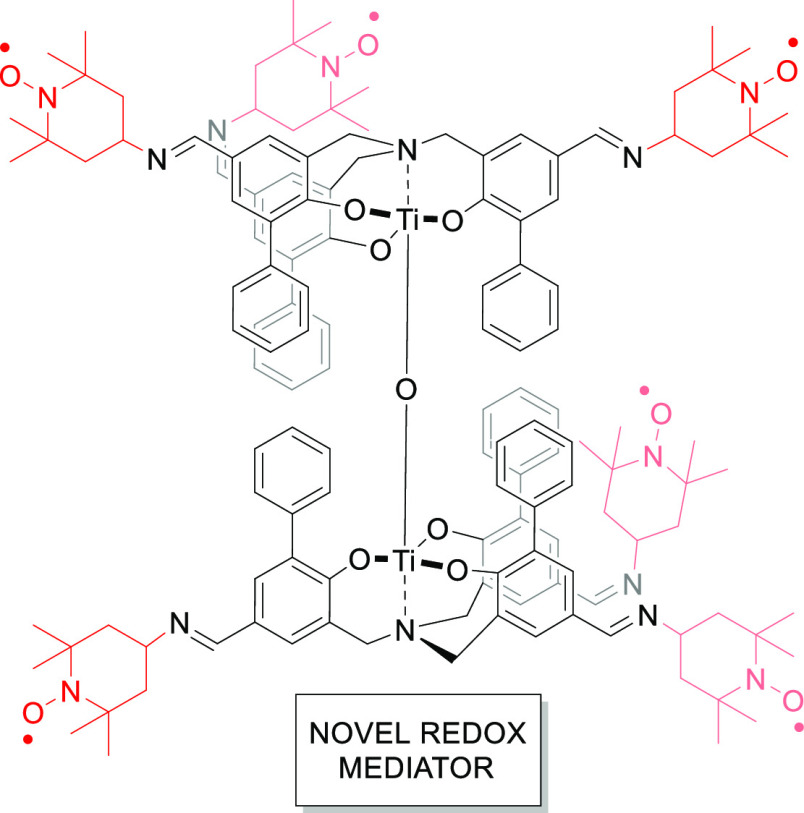

The spin–spin interactions between unpaired electrons in organic (poly)radicals, especially nitroxides, are largely investigated and are of crucial importance for their applications in areas such as organic magnetism, molecular charge transfer, or multiple spin labeling in structural biology. Recently, 2,2,6,6-tetramethylpiperidinyloxyl and polymers functionalized with nitroxides have been described as successful redox mediators in several electrochemical applications; however, the study of spin–spin interaction effect in such an area is absent. This communication reports the preparation of a novel family of discrete polynitroxide molecules, with the same number of radical units but different arrangements to study the effect of intramolecular spin–spin interactions on their electrochemical potential and their use as oxidation redox mediators in a Li–oxygen battery. We find that the intensity of interactions, as measured by the *d*_1_/*d* electron paramagnetic resonance parameter, progressively lowers the reduction potential. This allows us to tune the charging potential of the battery, optimizing its energy efficiency.

## Introduction

The spin–spin interactions between unpaired electrons in organic diradicals and polyradicals are of crucial importance in many areas such as organic magnetism,^1−5^ molecular charge transfer,^6^ or multiple spin labeling in structural biology.^7^ When unpaired electrons are in close proximity, the dominant interaction is likely to be spin-exchange coupling and dipole–dipole interactions. The origin of such radical–radical interactions could be intra- and/or intermolecular, that is, between radicals of the same molecule or radicals from different species. Intramolecular interactions exist when radical units within a structure are close enough and are detected at both low and high concentrations, whereas intermolecular interactions exist only at high concentrations because of the higher proximity between molecules. Among organic radicals, nitroxides have the advantage of being stable under ambient conditions and can be easily synthesized, functionalized, and manipulated. Di- and polynitroxides have shown improved properties with respect to mononitroxides as organic ferromagnets, labels in electron magnetic resonance imaging, radiation protectors during whole brain radiotherapy, or as polarizing agents in dynamic nuclear polarization (DNP).^8^ For example, when used as electron spin agents for DNP, dinitroxides can enhance the sensitivity of nuclear magnetic resonance signals by orders of magnitude compared with mononitroxides. Among other factors, the intramolecular exchange interactions and electron–electron dipolar coupling are the possible spin relaxation enhancement pathways.^9−11^ We are not aware of the effects reported beyond magnetic properties. Nitroxide radicals also have relevant electrochemical properties and have been used as redox charge mediators.

Redox mediation is a mechanism ubiquitous in nature, used to transport electrons in solution phase, usually to connect a catalytic center to another one or to a reactant. In general, the redox mediator (RM) is a soluble component able to exchange an electron with a redox center, to diffuse to a different redox center, and to exchange again an electron to restore its initial state. In the cell respiratory system, for example, nicotinamide adenine dinucleotide hydrogen (NADH) shuttles electrons through the membrane, and three distinct complexes are involved in the electron transport chain in the mitochondria before reducing oxygen to water.^12,13^ In photosynthesis, different quinones play similar roles between the reactive complexes involved.^14^ In part, inspired by such systems, several chemical and electrochemical energy conversion or storage systems rely or are improved by the use of RMs (i.e., artificial photosynthesis, organic dye-sensitized solar cells, pseudocapacitors, and redox flow, lithium–sulfur, and metal–oxygen batteries).^15^ Nitroxides, in particular, 2,2,6,6-tetramethylpiperidinyloxyl (TEMPO) monoradical, have been widely studied in the past two decades in energy storage,^16,17^ either as part of the cathode^18^ or in the electrolyte.^19^ Being part of the cathode, TEMPO could be used, for instance, as an active material in an organic-based paper battery^20^ or to tune conductivity in a conjugated radical polymer battery.^21,22^ On the other hand, it could be used in the electrolyte as a catholyte for redox flow batteries^23^ or as a soluble oxidation mediator in metal–oxygen batteries.^24^ This wide use is due to its appropriate potential, kinetics, and availability.^25^ Some polynitroxide compounds, such as polymers functionalized with nitroxides, have been described exhibiting a high mediation of charge.^26,27^

**Scheme 1:**
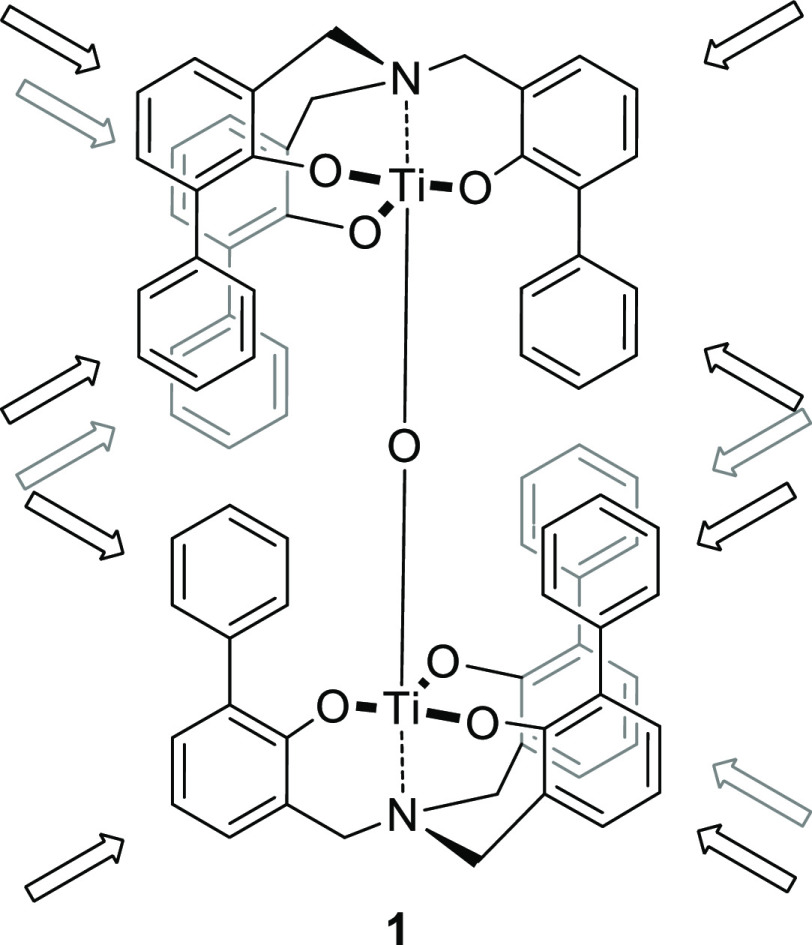
Reported μ-Oxo Dinuclear Titanium Complex **1** with the Possible Functionalization Indicated

The aim of this study is to functionalize the molecules in Scheme 1 to obtain a polynitroxide. To the best of our knowledge, mediation has not been reported with discrete molecules presenting several identical (nitroxides) redox centers with a well-defined arrangement, and the effect of intramolecular radical interactions still remains a challenging mechanism to understand. In addition, a comparison between polynitroxides and mononitroxides is also absent. The intensity of the intramolecular spin–spin interactions increases mainly with the proximity of the radical units and the number of interacting radicals and can be detected and studied by electron paramagnetic resonance (EPR) spectroscopy.^28,29^ In this work, we study the effect of intramolecular spin–spin interactions in polynitroxide molecules on their electrochemical potential and their use as RMs, in particular as charge mediators in aprotic lithium–oxygen batteries. These batteries present high theoretical capacities because of the reaction between pure lightweight elements (Li and O_2_) and the solid product (Li_2_O_2_) but are affected by several issues related to the formation of reactive intermediates and passivation by Li_2_O_2_.^30^

Mediators are critical in two different processes of metal–air batteries: they allow to delay electrode passivation during discharge and assist peroxide removal during charge.^31,32^ Using mediators with the redox potential below the oxygen reduction potential (*E*^0^ = 2.96 V vs Li/Li^+^) results in an oxygen reduction reaction (ORR) with enhanced kinetics and discharge capacity.^33−36^ Instead, when its redox potential is above the equilibrium potential, the mediator is active during charge, where it is oxidized at the electrode, diffuses to Li_2_O_2_, with which it chemically reacts to give place to oxygen evolution (oxygen evolution reaction OER).^24,31,37−40^ Thus, in the case of TEMPO nitroxide, we have the following catalytic scheme
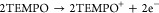

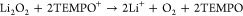
1

For efficient operation, apart from the appropriate reduction potential, a mediator requires stability in cell components and intermediates,^39^ reactivity with Li_2_O_2_,^41^ and moderate diffusivity.^32^ The latter is necessary to minimize shuttling to the anode and is favored by larger compounds, whereas nitroxides are regarded as one of the most stable functional groups among halogenides, quinones, and several other organic molecules.^42^

To obtain the target molecules, the aim of the present study, we have used as scaffold triphenolamines,^43^ in particular a μ-oxo dinuclear titanium complex **1** (Scheme 1).^44^

In recent years, we reported about the use of tetradentate metal complexes in catalysis,^45−48^ molecular recognition,^49−54^ and as molecular scaffolds for multiple functionalization. Among the different structures, μ-oxo dinuclear titanium complex **1** represents the ideal architecture to become a scaffold for multiple functionalization (Scheme 1). This system, which spontaneously forms starting from two titanatrane units via selective hydrolysis, has a well-defined geometry, and it is stable even in the presence of water. The overall stability, combined by the defined geometry, makes this molecular structure ideal for multiple functionalization with RMs.

In the present paper, we report about: (i) the synthetic evolution of the system in order to obtain a molecular scaffold suitable for multiple functionalization, (ii) the preparation of defined molecular systems containing the same number of radical units with different arrangements, some with closer and others with more distant radical dispositions, (iii) the study of the intramolecular interaction strength among radicals in the different arrangements, and (iv) the effect of such radical interactions on their electrochemical behavior and RM capabilities.

## Results and Discussion

### Synthesis of Multiple Radical Molecular Architectures

**Scheme 2:**
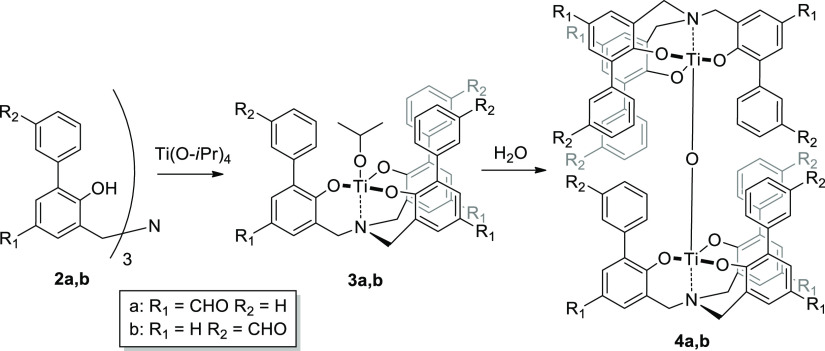
μ-Oxo Complexes **4a–b** can be Obtained from the Corresponding Phenol Derivatives **2a–b** by Reaction with Ti(O-*i*Pr)_4_; the Mononuclear Titanatrane Systems **3a–b** Evolve Spontaneously to the Corresponding **4a–b** in the Presence of Traces of water

As shown in our previous paper, titanatranes with bulky phenyl substituents in ortho positions spontaneously form highly stable μ-oxo dinuclear complexes in the presence of traces of water.^44^ We took advantage of this chemistry to synthesize a series of novel μ-oxo complexes **4a–b**, bearing aldehydes in different positions (Scheme 2). Aldehydes were chosen as possible anchoring points for the subsequent introduction of amino-TEMPO units via the imine condensation reaction. The parent ligands **2a–b** were prepared with a newly developed synthesis method (see Supporting Information, Chapter S1).

This takes advantage of either a direct threefold formylation of a preformed ligand, for the functionalization of the phenol para positions (*viz.***2a**), or a Suzuki coupling with formyl-arylboronic acid for the functionalization of the upper substituted phenyl ring (*viz***2b**). The reaction between the amine triphenolates **2a–b** and Ti(O-*i*Pr)_4_ resulted in the *in situ* formation of the *C*_3_ mononuclear Ti(IV) complexes **3a–b** which rapidly and spontaneously self-assemble, in the presence of traces of water, into the dinuclear μ-oxo **4a–b**, as the only *S*_6_-symmetric system (Scheme 2). The complexation and formation of the dinuclear μ-oxo titanium (IV) complexes can be easily followed by ^1^H NMR spectroscopy. As an example, by the addition of 1 equiv of Ti(O-*i*Pr)_4_ to **2b**, the ^1^H NMR spectrum in CDCl_3_ shows the formation of a single set of signals at 3.69 ppm for the methylene protons in α to the nitrogen of **3b** (see Supporting Information Figure S12). Upon the addition of few amount of water, **3b** rapidly evolves into the dinuclear μ-oxo complex **4b**, which precipitates over time from the solution. The ^1^H NMR spectrum of **4b** shows the formation of an AB system at 4.40 and 3.31 ppm, corresponding to the methylene protons (see Supporting Information Figure S13). Moreover, the aromatic protons of the peripheral aryl rings, together with the aldehydic signal, are shifted upfield (for CHO, from 9.94 to 9.20 ppm) because of the intercalation of the rings around the μ-oxo bridge. The formation of the dimeric complexes **4a–b** is also confirmed by electrospray ionization-mass spectrometry (ESI-MS) analysis. As an example, for **4b**, the spectra, both in the positive and negative modes, clearly display the characteristic isotopic distribution for the formation of [M]^−^ (*m*/*z* = 1041.2) or of the complex having Na^+^ counterion (*m*/*z* = 1423.4).

**Scheme 3:**
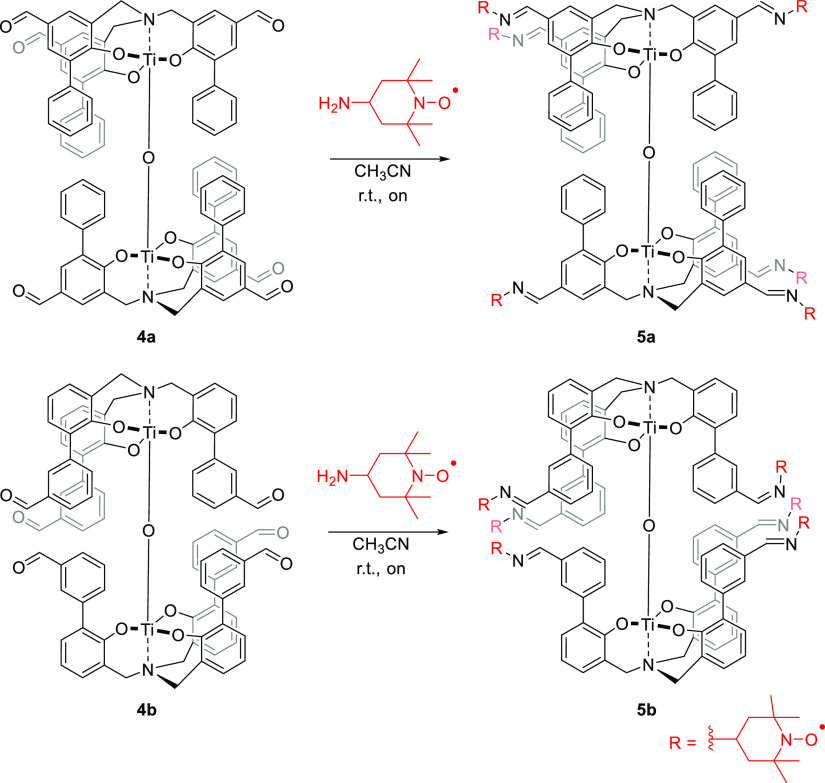
Synthesis of TEMPO-Functionalized Dinuclear μ-Oxo Titanium (IV) Complexes **5a–b**

**Scheme 4:**
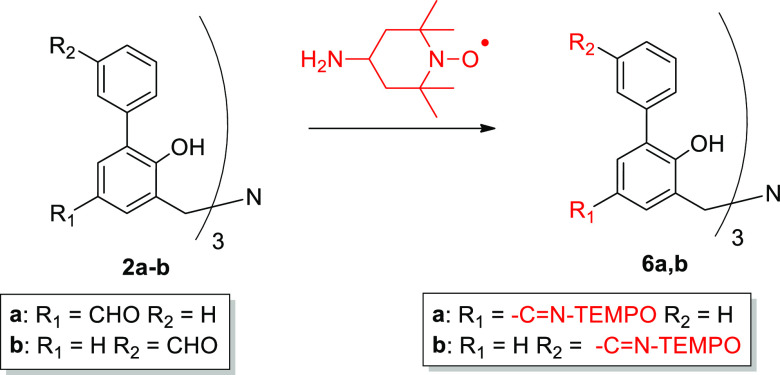
Synthesis of TEMPO-Functionalized Ligands **6a–b**

Condensation between **4a–b** and the radical 4-amino-TEMPO has allowed to obtain compounds **5a–b** (Scheme 3). The handling of radicals in solution was carried out under dark and anhydrous conditions to avoid, respectively, the possible degradation of the radicals and imine bond hydrolysis. This postfunctionalization leads to the construction of stable and spatially ordered structures with multiple mediator functionalities disposed in a controlled way into space. Similarly, to have a comparison with a less spatially defined system, radical carriers **6a–b** were prepared starting from ligands **2a–b** (Scheme 4). The novel μ-oxo complexes 5**a–b** and ligands **6a–b** were characterized by ESI-MS, Fourier transform infrared (FTIR) spectroscopy, elemental analysis, and EPR spectroscopy. In the ESI-MS spectra, all experimental isotopic clusters were in agreement with the theoretical ones. The FTIR measurements for all the systems showed the disappearance of the characteristic carbonyl stretching of the aldehyde (at *ca*. 1700 cm^–1^) and the appearance of the stretching peak of the C=N bond (at *ca*. 1600 cm^–1^). The full functionalization with the radicals of all compounds was quantitatively determined by EPR spectroscopy (see Supporting Information Table S1).

**Figure 1:**
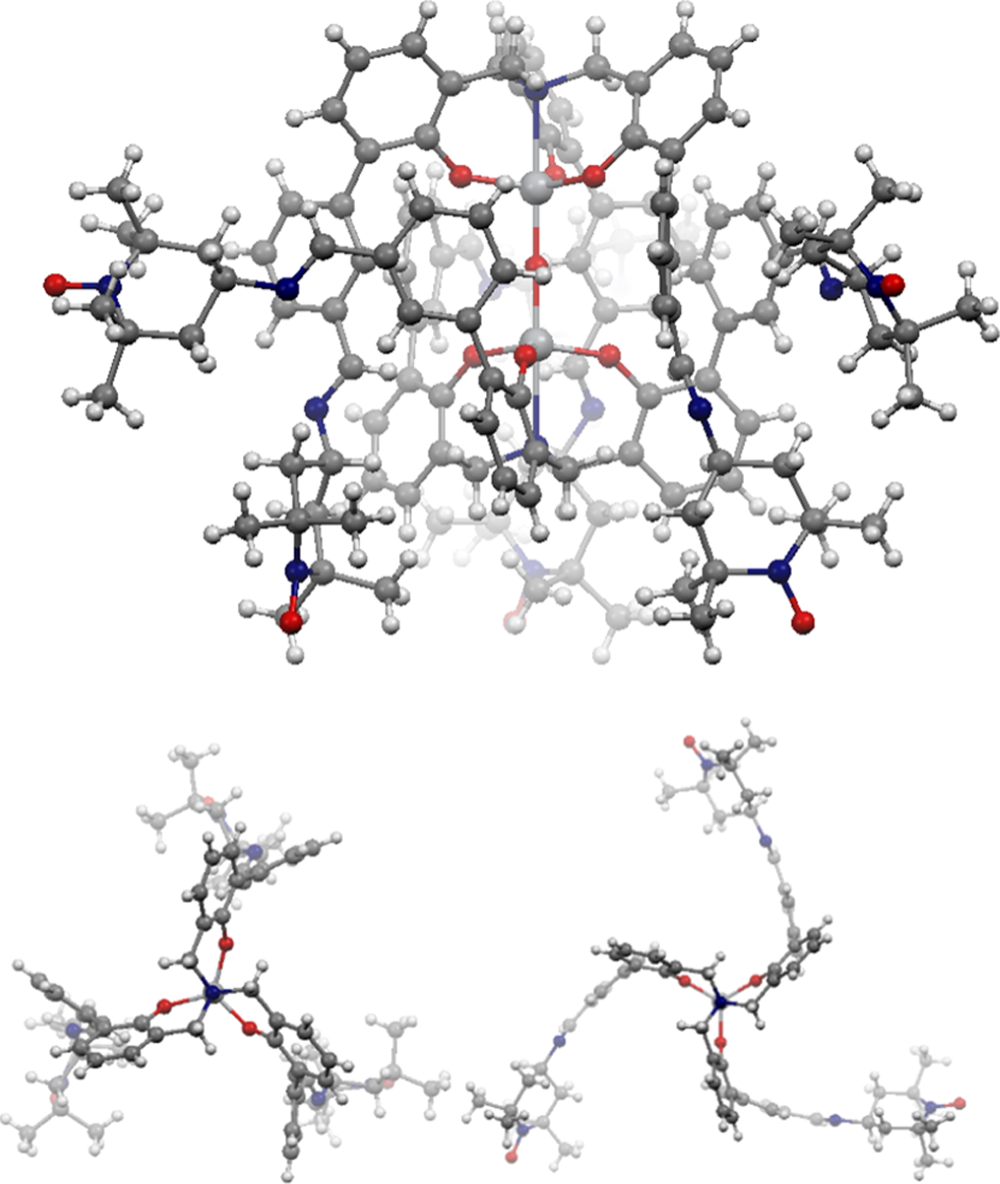
X-ray single crystal structure of **5b** and details of the relative orientations of the two titanatrane units in the μ-oxo system.

The X-ray structure of **5b** unambiguously confirmed the dimer formation as well as the condensation between the radical 4-amino-TEMPO and the aldehyde moieties present in **4b** (Figure 1). The molecular structure of this radical-functionalized dinuclear system **5b** shows a *C*_3_ symmetry in the solid state instead of the *S*_6_-symmetric system in solution that was observed for **4b**.

Ligands **6a–b** and the corresponding μ-oxo complexes **5a–b** were investigated by EPR to gather information on the relative arrangement of the radicals in solution. The results obtained were then correlated to their electrochemical properties evaluated by cyclic voltammetry and finally to the OER RMs for Li–O_2_ batteries. The same concentration has been used in each pair.

#### EPR Spectroscopy for Polyradical Species **5a–b** and **6a–b**

**Figure 2:**
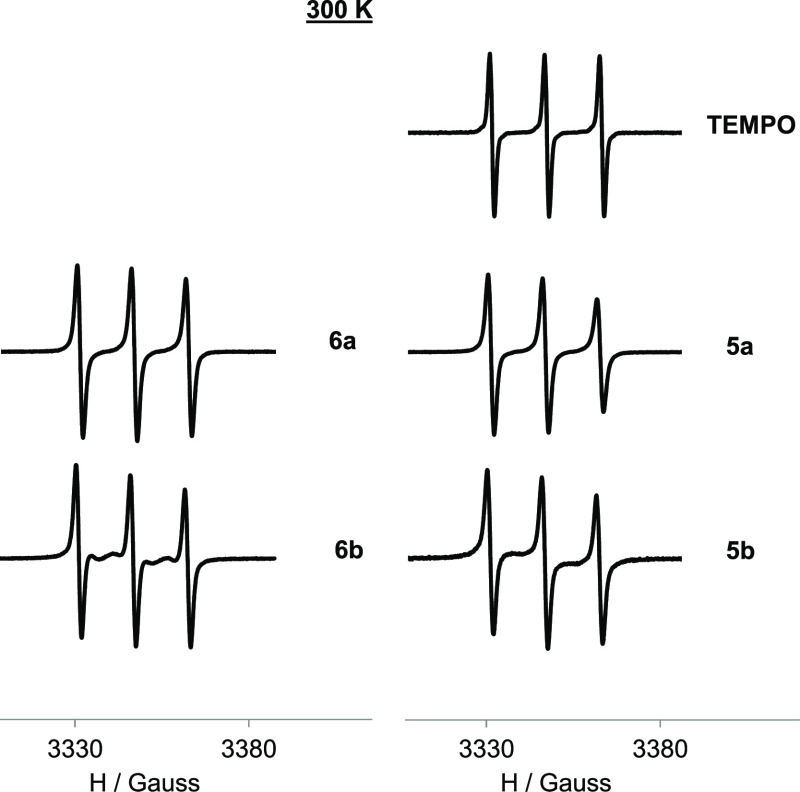
Normalized EPR spectra of TEMPO, ligands **6a–6b,** and μ-oxo complexes **5a–5b** in DCM/toluene 1:1, at 300 K and 1 mM.

The EPR study was done in diluted conditions to focus on intramolecular radical interactions. The EPR spectra of the polyradical species at 300 K (Figure 2) showed mainly a three-line pattern like that of the TEMPO free radical, although with broader lines and a selective decrease of the high-field line because of the hindered motion of the radicals attached to a big molecule. In addition, some signs related to the radical interactions were observed. In the EPR spectra of the corresponding **b** species (**6b** and **5b**), some little alternating linewidth effect compared with their respective **a** species (**6a** and **5a**) was observed. This means some spin-exchange interaction among the radicals in such compounds. In fact, the radicals in **b** conformations present more degrees of freedom than in **a**, favoring their mobility and hence their proximity.

**Figure 3:**
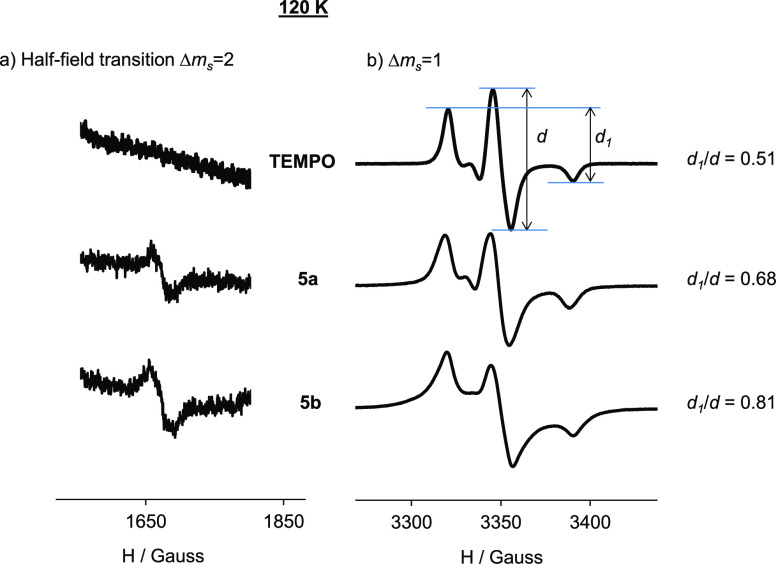
(a) |Δ*m*_s_| = 2 transition at half-field EPR spectra and (b) |Δ*m*_s_| = 1 EPR spectra of TEMPO and μ-oxo complexes **5a–5b** in DCM/toluene 1:1 at 120 K and 1 mM.

In a frozen solution, 120 K, the EPR spectral shape changes completely. Under these anisotropic conditions, the spectral shape is sensitive to the distance between neighboring nitroxides up to ca. 2 nm, and a convenient measure of the strength of the dipole–dipole interactions is therefore given by the empirical ratio of peak heights, the *d*_1_/*d* value (Figure 3).^55^ The higher the ratio, the shorter the distance between the radical centers, and hence the higher the radical interactions. Table 1 displays the calculated *d*_1_/*d* values for all compounds from their corresponding frozen solution spectra (shown in Figures 3 and Supporting Information S15–S16). The *d*_1_/*d* ratio was 0.51 for the monoradical TEMPO where intramolecular interactions are absent, whereas for all the polyradical species, this ratio was much higher. This suggested that all of them presented intramolecular dipolar interactions and that some of their radicals should be located at distances lower than 2 nm. This compares well with the 7.846 Å radical–radical distance found in the crystal structure. In detail, both in the ligands and μ-oxo complex pairs, higher *d*_1_/*d* values, that is, closer radicals, in their corresponding **b** arrangement (0.85 and 0.81 for **6b** and **5b** as against 0.69 and 0.68 for **6a** and **5a**, respectively) were observed. In addition, under these conditions, all polyradical compounds showed a half-field transition of |Δ*m*_s_| = 2. This signal is characteristic of dipolar coupled spins and a direct evidence of the presence of a high-spin state. It is mainly generated by the presence of two radical units closer than a critical distance, and its intensity depends on the average distance between them and the number of interacting pairs of radicals. Therefore, the half-field signal intensity was used as a second independent parameter to quantify the radical interactions in our compounds. In Table 1 are also reported the normalized half-field signal intensities of all compounds, and in Figures 3 (and Supporting Information S17), their corresponding EPR spectra.

**Table 1:** EPR and Electrochemical Parameters of Compounds **5a–b** and **6a–b**^a^

	*d*_1_/*d*	|Δ*m*_s_| = 2 intensity	*E*_1/2_ (V) vs Ag/AgCl	voltage (V) vs Li^+^/Li at 0.05 mA/cm^2^
**6a**	0.69	1.0	0.86	3.94
**6b**	0.85	1.05	0.84	3.83
**5a**	0.68	1.3	0.86	3.89
**5b**	0.81	1.6	0.83	3.81

a Measure replicates show variations in the range of ±2 mV for *E*_1/2_ and ±0001 for *d*_1_/*d*.

Following the same trend, the half-field intensity was also larger in the **b** species. This difference in intensity is significant in the μ-oxo complex pair (**5a** vs **5b**) and can be explained taking into account the different locations of the radicals in the scaffold. In **5b,** the radicals of both titanatranes are disposed in closer proximity than in **5a**. In fact, the **5b** X-ray structure displays three close enough (7.846 Å) pairs of radicals (each pair with the radicals of both titanatranes). The corresponding EPR study of the TEMPO free radical in frozen solution is explained in the Supporting Information.

#### Cyclic Voltammetries of Compounds **5a–b** and **6a–b**

**Figure 4:**
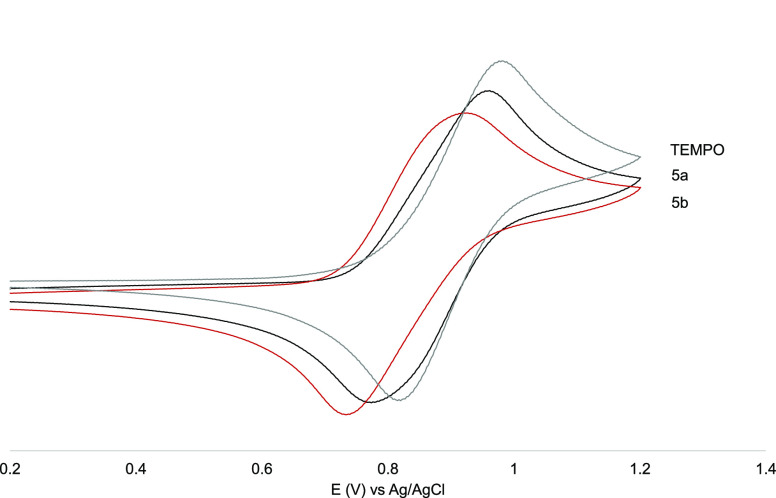
Cyclic voltammetry of TEMPO and μ-oxo complexes **5a–5b** at 1 mM in DMF with 0.1 M TBAHFP *vs* Ag/AgCl at the scan rate of 200 mV/s.

**Figure 5:**
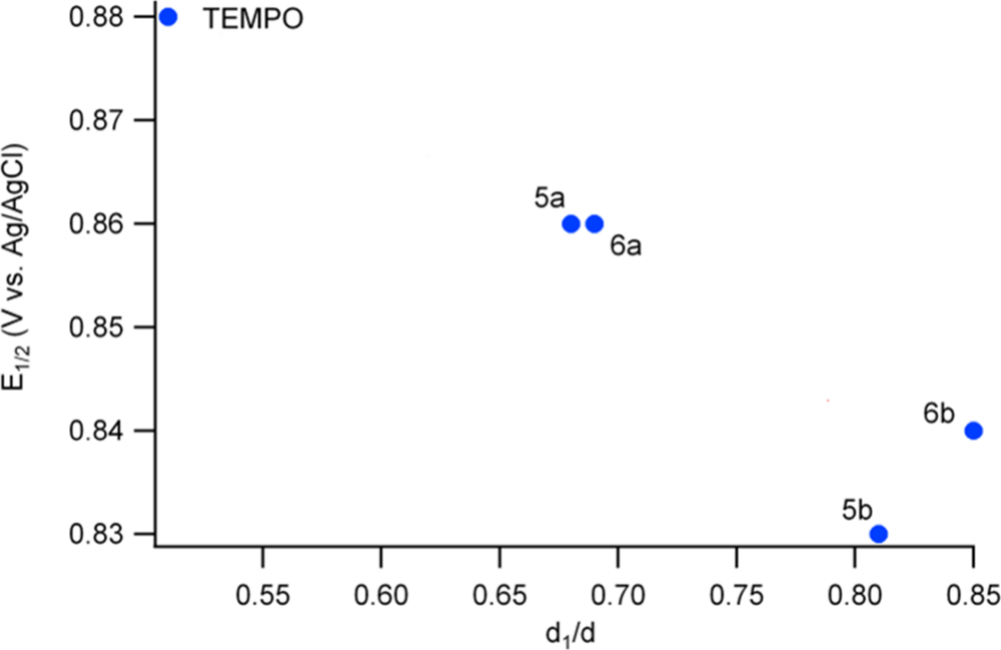
Half-wave potential *E*_1/2_*vs d*_1_/*d* of TEMPO and polynitroxides **6a–b** and **5a–b** at the same molecular concentration of 1 mM.

The electrochemical properties of the polyradical species under study and the TEMPO free radical were evaluated by cyclic voltammetry (CV) in DMF, with 0.1 M of tetrabutylammonium hexafluorophosphate (TBAHFP) as the electrolyte. The corresponding cyclic voltammograms are shown in Figure 4 (see also Supporting Information Figure S18–S20 and Figure S21 for CV at different scan rates), and the half-wave potential values E_1/2_ are included in Table 1. The polyradical compounds exhibited a reversible redox wave at lower potential values than the monoradical TEMPO (*E*_1/2_–0.9 V, see Supporting Information Table S2). Focusing on the different polynitroxides, it can be observed that the **b** species (**6b** and **5b**) exhibited lower *E*_1/2_ potential than their corresponding homologues with the **a** arrangement (**6a** and **5a**, respectively). These relations are a clear indication of the mutual interactions between the redox centers existing in these polyradical species (absent in the monoradical TEMPO) and can be explained by means of intramolecular electron–electron interaction effects. As previously reported,^56^ in the polyradical species with a closer radical disposition (higher interactions), this shift was higher. As shown in Figure 5 we observe an interesting correlation between the *E*_1/2_ potential and the *d*_1_/*d* parameter. This suggests that the same interactions caused by the close distance forced by the ligand geometry on one hand are reflected in the magnetic radical coupling, and on the other hand destabilize the electronic level of the radical state, resulting in a lower reduction potential.

#### Compounds **5a–b** and **6a–b** as Redox Charge Mediators

**Figure 6:**
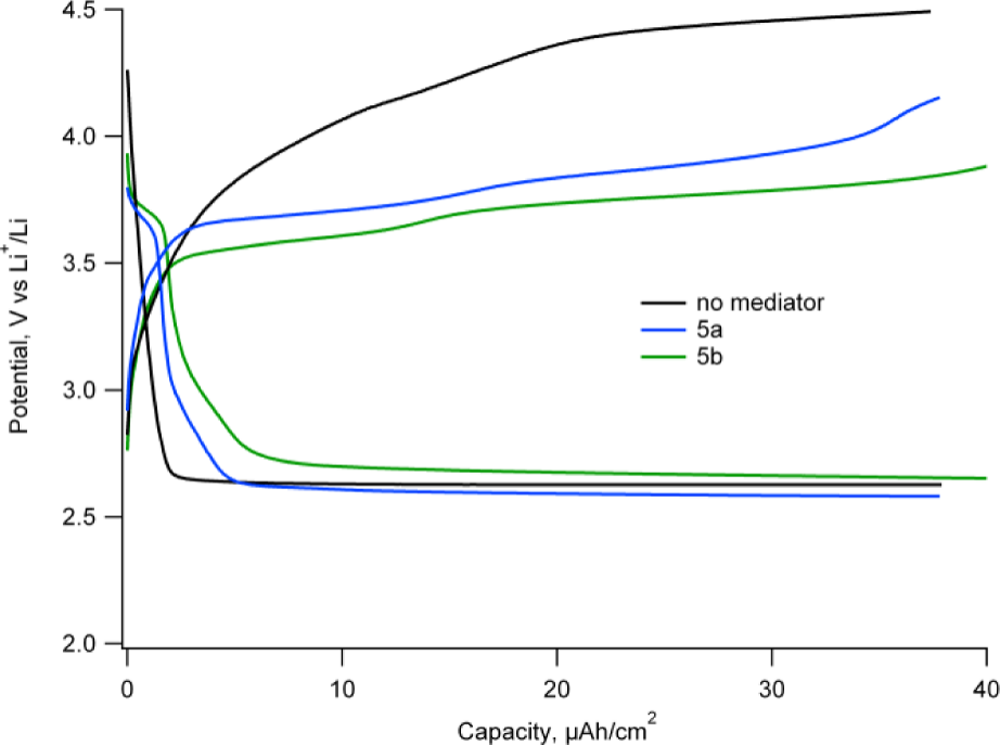
Charge and discharge galvanostatic pulses with the electrolyte without additives and using **5a–b** mediators at 1 mM at current densities of 0.05 mA/cm^2^.

Ligands **6a–b** and their corresponding μ-oxo complexes (**5a**–**b**) were tested as redox charge mediators for Li–O_2_ batteries. Typical galvanostatic discharge and charge profiles are reported in Figure 6 (see also Supporting Information Figure S22 for the complete dataset and the corresponding polarization curves). Table 1 reports their corresponding cell voltages *versus* Li^+^/Li when a charging current of 0.05 mA/cm^2^ was applied. Compared to the electrolyte without additives, we clearly observe mediation of charge, with general tendencies consistent with the potential shifts obtained by cyclic voltammetry, as graphically also shown in Figure S24. This implies that the larger molecular size does not affect the mediation activity as much as the redox potential.

Remarkably, we can observe that by using TEMPO mononitroxide at different concentrations, from 1 to 12 mM, we measured systematically higher charge voltages than those obtained with the polynitroxide species under study (Supporting Information Figure S23 and Tables S2–S3). Although there was a tendency toward lower charge voltage by increasing the TEMPO radical concentration, which was also reported elsewhere,^25^ even at double radical concentration than polynitroxides (12 mM), the TEMPO charge voltage was still higher (see Supporting Information Table S2). In addition, the charge potential decreases from **6a** to **5b,** much more than the corresponding E_1/2_ variation even if the same radical concentration has been used. This suggests that intramolecular interactions also have a direct impact on the kinetic efficiency of polynitroxides as charge mediators, which sums to the thermodynamic variation of redox potential.

## Conclusions

Triphenolamines and μ-oxo dinuclear Ti(IV) complexes with versatile multiple functionalization and a well-defined geometry have been synthesized and characterized. These molecular scaffolds have permitted us to anchor up to six redox-active TEMPO radical units in two different arrangements (some with closer and others with more distant radical dispositions) to study the influence of the intramolecular radical interactions on their electrochemical and OER mediator behavior. These polyradical species have been synthesized and characterized. We studied by EPR and X-ray diffraction the arrangement of the radical units in such molecular scaffolds, and their mutual interactions, and quantified their electrochemical behavior by cyclic voltammetry and their radical efficiency as RM, by the charge voltage in a Li/O_2_ battery.

We conclude that multiple TEMPO redox units in the same discrete molecular scaffold can favor the efficiency as OER mediator compared with monoradical species. In particular, the better performances observed are related to the closer disposition of the radical units and the higher number of pairs of radicals that can interact intramolecularly. Such intramolecular interactions seem to decrease the half-wave potential of the electroactive TEMPO radical units. In a Li–O_2_ battery, this allows to tune the charging potential toward lower values, making more efficient the OER RM process. In fact, the smaller difference between the discharge and charge potential increases the energy efficiency, and the lower overpotential decreases the probability of secondary reactions. Thus, this study suggests a correlation between the radical efficiency as RM and the intramolecular radical interactions quantified by EPR. Further studies are in progress to evaluate such effects with a bigger family of polynitroxide systems.

## Supporting Information Available

Compound synthesis and characterization, EPR, and charge and discharge galvanostatic pulses (PDF)
