# Supplementary material for: Organic Polyradicals as Redox Mediators: Effect of Intramolecular Radical Interactions on Their Efficiency
Source: ACS Appl Mater Interfaces. 2020 Sep 15;12(41):45968–75. doi: 10.1021/acsami.0c09386 (PMC8011802; doi:10.1021/acsami.0c09386)
Supplement: Supplementary file 1 — am0c09386_si_001.pdf [file am0c09386_si_001.pdf]

Organic PolyRadicals as Redox Mediators: Effect of Intramolecular Radical Interactions in their Efficiency.

Elena Badetti,[†,≠,*] Vega Lloveras,[‡] Emanuele Amadio,[†] Rosalia Di Lorenzo,[†] Mara Olivares-Marín,[‡] Alvaro Y. Tesio,[#] Songbai Zhang,[‡] Fangfang Pan,[%,&] Kari Rissanen,[%] Jaume Veciana,[‡] Dino Tonti,[‡,*] Jose Vidal-Gancedo,[‡,*] Cristiano Zonta,[†] Giulia Licini[†,*]

† Department of Chemical Sciences and CIRCC, Padova Unit, via Marzolo 1, 35131 Padova (Italy) ‡ Institut de Ciència de Materials de Barcelona (ICMAB-CSIC), campus universitari de Bellaterra, E-08193, Cerdanyola del Vallès (Spain) and Networking Research Center on Bioengineering, Biomaterials and Nanomedicine (CIBER-BBN), E-08193. Barcelona (Spain). # Centro de Investigación y Desarrollo en Materiales Avanzados y Almacenamiento de Energía de Jujuy (CIDME-Ju), Centro de Desarrollo Tecnológico General Manuel Savio, Av. Martijena S/N, Palpalá Y 4612 - Jujuy, Argentina. % University of Jyvaskyla, Department of Chemistry, P. O. Box 35, 40014 Jyväskylä, Finland. ≠ Present address: DAIS—Department of Environmental Sciences, Informatics and Statistics, University Ca' Foscari of Venice, Via Torino 155, 30172 Venice Mestre, Italy. & Present address: Central China Normal University, Key Laboratory of Pesticide & Chemical Biology of Ministry of Education, Hubei International Scientific and Technological Cooperation Base of Pesticide and Green Synthesis, College of Chemistry, Luoyu Road 152, Wuhan 430079, People's Republic of China

Index Contents

1. Materials and methods S1
2. Synthesis S2
3. NMR spectra S10
4. Quantitative EPR study S17
5. Frozen solution EPR spectra S18
6. Half-field EPR spectra S19
7. EPR study of monoradical TEMPO S20
8. Cyclic voltammetries S20
9. Charge and discharge galvanostatic pulses S23
10. Charge and discharge plateau voltage (vs Li^+/Li) S24
11. X-ray Crystallography S26

1. Materials and methods

Chemicals and dry solvents were purchased from Sigma Aldrich and used without further purification. Where "degassed" solvents or solutions are noted, degassing was carried out by three freeze-pump-thaw cycles. Flash chromatography (FC) was performed as described in literature[1] using Macherey-Nagel silica gel 60 (0.04-0.063 mm, 230-400 mesh). TLC analyses: Macherey-Nagel POLYGRAM® SIL G/UV_{254}; detection by UV/VIS and by treatment with PMA staining reagent made from a solution of phosphomolibdic acid ($H_3PMo_{12}O_{40}$, 10 g) in 100 mL ethanol. ^1H and $^{13}C\{^1H\}$-NMR spectra were recorded at 301K on Bruker AC-200, 300 MHz instruments. Chemical shifts (δ) have been reported in parts per million (ppm) relative to the residual undeuterated solvent as an internal reference. The following abbreviations have been used to explain the multiplicities: *s* = singlet, *d* = doublet, *t* = triplet, *dd* = doublet, doublet, *m* = multiplet, *br* = broad. $^{13}C\{^1H\}$-NMR spectra have been recorded with complete proton decoupling. ESI-MS spectra have been obtained on: i) a LC/MS Agilent series 1100 spectrometer in both positive and negative modes using acetonitrile/formic acid 0.1% as mobile phase, with ESI-ion trap mass detector or a ii) ESI-TOF Mariner™ Biospectrometry™ Workstation of Applied Biosystems by flow injection, using acetonitrile or methanol/formic acid 0.1% as mobile phase, was used. Analytical gas chromatography with mass spectrometry detector (GC-MS) has been carried out on an Agilent 6850 spectrometer equipped with a split mode capillary injector and electron impact mass detector. Injector temperature has been set to 250 °C, detector temperature has been set to 280 °C and the carrier gas is He (1 mL/min) with a HP-5MS column. Melting points are uncorrected and have been determined with a Leitz-Laboroux 12. IR spectra have been recorded on a Nicolet 5700 FT-IR, with range 4000-400 cm^{-1} and resolution 4 cm^{-1}, using KBr pellets or NaCl plates.

EPR spectra were obtained with an X-Band Bruker ELEXSYS E-500 spectrometer equipped with a TE102 microwave cavity, a Bruker variable temperature unit, a field frequency lock system Bruker ER 033 M and a NMR Gaussmeter Bruker ER 035 M. The modulation amplitude was kept well below the line width, and the microwave power was well below saturation. All samples were previously degassed with Ar. The same concentration was used in each pair. For 5a and 5b, 1 mM, and for 6a and 6b, either 1 mM or 2 mM, obtaining the same trend in both cases.

The CV measurements were done with a potentiostat Autolab/PGSTAT204 Metrohm in a standard 3 electrodes cell. Compounds were dissolved in 0.1 M of TBAHFP as electrolyte in DMF and using a glassy carbon electrode as the working electrode, a Ag/AgCl reference electrode and a platinum wire as the auxiliary electrode. The same concentration was used in each pair. For 5a and 5b, 1 mM, and for 6a and 6b, either 1 mM or 2 mM, obtaining the same trend in both cases.

Li-O_2 batteries were 2-compartment cells, separated by a lithium-ion conducting glass-ceramics (LICGC™ AG-01, Ohara Corporation) soldered on a polypropylene support. The cathode consisted of carbon black (Super P, Timcal) supported on a 0.79 cm^2 carbon paper (Toray TGP-H-060). It was prepared by mixing 90% Super P with 10 wt% of polyvinylidene difluoride in N-methylpyrrolidone. The slurry obtained was used to impregnate the carbon paper surface on both sides, which was finally dried at 100 °C for 12 h and punched in disks of 10 mm diameter. The electrolyte was a solution of 1M lithium trifluoromethanesulfonate (99.9%, vacuum-dried overnight) in anhydrous diethylene glycol dimethyl ether (DEGDME, ≥99 %). For the cathode side TEMPO redox species were added to the same solution in order to obtain the specified

concentrations and stored protected from light. The same concentration was used in each pair, and in addition, the same concentration per radical unit among them. 1 mM for 5a and 5b and 2 mM for 6a and 6b. The final electrolyte water content resulted of <30 ppm as determined by a Coulometric Karl Fischer titrator (Metrohm KFC 899).

The battery casing consisted of a homemade teflon design based on the cell described by Bender et al.[2] assembled in an Ar-filled glove box with < 1 ppm moisture. The inner cylinder was filled with several layers: 0.1 mm Nickel foil (99% purity, ADVENT Research Materials), 10 mm diameter as current collector; two layers of lithium metal foil as anode (Rockwood Lithium, 0.3 mm tick); two disks made by glass microfiber filter (90 mm thick, Prat Dumas), soaked with 100 mL of additive-free electrolyte; the glass-ceramic separator; two glass microfiber disks soaked with 100 mL of mediator electrolyte; the carbon cathode, and finally two stainless steel meshes (AISI 316, 180 mesh per inch, ADVENT Research Material Ltd). To replace Ar with O_2 the cell was purged with pure oxygen for a few minutes.

Galvanostatic discharge/charge pulses were performed between 0.02 and 1 mA/cm^2 with a cutoff capacity of 40 $\mu A/cm^2$ by using a MTI BST8 multichannel potentiostat.

2. Synthesis

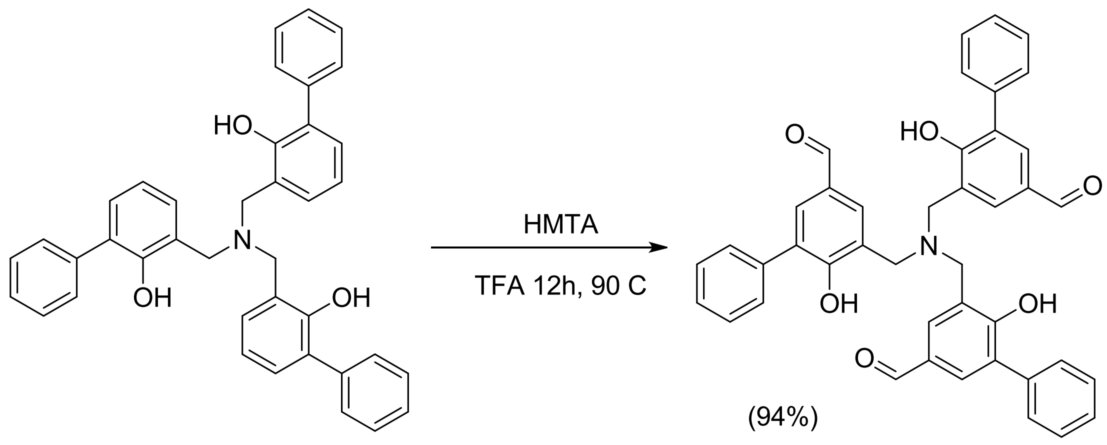

1a

Synthesis of Tris-(2-hydroxy-3-benzyl-5-formylbenzyl)amine (**1a**). A nitrogen purged Schlenk tube was charged with *ortho* substituted *Tris-(2-hydroxy-3-benzyl)amine* (2.16 g, 4.03 mmol) and hexamethylenetetramine (3.39 g, 24.2 mmol) and diluted with TFA (3 mL/mmol sub.) and the mixtures were stirred at 90°C for 12h. The crude mixtures were evaporated to dryness and HCl_{aq} 4N (36 mL) was added and the mixture was stirred for 3 h at 90 °C. The precipitate was filtered off, washed with $NaHCO_3$ and extracted with CH_2Cl_2. The organic phase was dried over anhydrous Na_2SO_4 and evaporated to dryness. The final product was obtained by crystallization in acetonitrile as a yellow solid (94%). ^{1}H-NMR (300 MHz, $CDCl_3$) δ = 9.80 (3H, *s*) 7.69 (*d*, *J* = 2 Hz, 3H), 7.66 (*d*, *J* = 2 Hz, 3H), 7.44-7.35 (15H, *m*), 4.08 (6H, *s*). $^{13}C\{^1H\}$-NMR (75.5 MHz, $CDCl_3$) δ = 190.75, 158.20, 135.80, 132.76, 131.59, 129.48, 129.33, 129.28, 129.23, 128.49, 124.01, 56.49. HRMS (ESI): *m/z* calc. 647.23; exp. 648.2390 $[M + H]^+$. IR (KBr) ν (cm^{-1}): 579, 616, 699, 735, 778, 805, 893, 983, 1030, 1092, 1151, 1317, 1387, 1425, 1475, 1497, 1585, 1694, 1808, 1891, 1957, 2730, 2831, 3055, 3349, 3521, 3643.

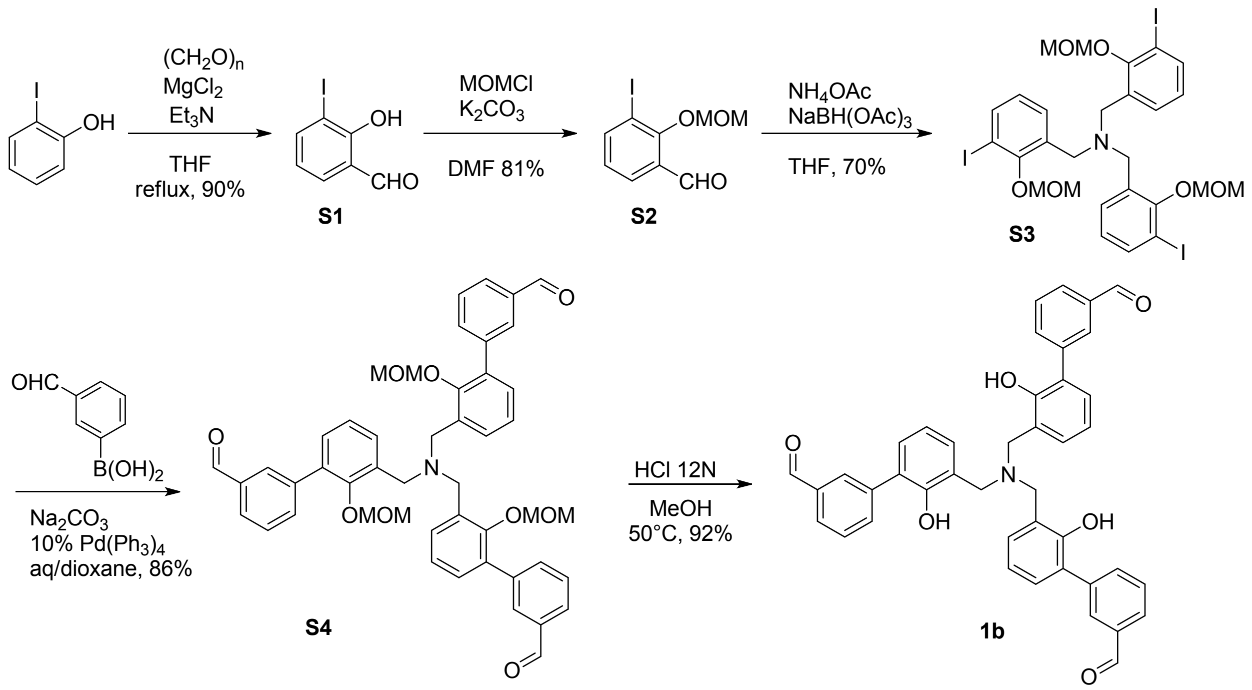

*Synthesis of 2-hydroxy-3-iodobenzaldehyde (**S1**):* To a stirred solution of 2-iodophenol (113.6 mmol, 25 g) in dry THF (200 mL) were added $MgCl_2$ (148 mmol, 14 g) and Et_3N (227.2 mmol, 31.7 mL) under a N_2 atmosphere. The mixture was stirred at room temperature for 30 min. Then, paraformaldehyde (568 mmol, 17 g) was added and the stirred suspension was heated to reflux. After 18 h the cooled reaction mixture was acidified with 1N HCl (100 mL), extracted with EtOAc, and washed with brine. The organic phase was dried over Na_2SO_4 and concentrated under vacuum. The crude product was purified by crystallization in *n*-hexane affording the final product as a yellow solid (90 %).^{1}H-NMR (300 MHz, $CDCl_3$) δ = 11.81 (s, 1H); 9.76 (s, 1H) 8.00 (dd, *J* = 1.6, 7.7 Hz, 1H), 7.56 (dd, *J* = 1.6, 7.7 Hz, 1H), 6.85 (t, *J* = 7.7 Hz, 1H). $^{13}C\{^{1}H\}$-NMR (75.5 MHz, $CDCl_3$) δ = 85.4, 120.4, 121.6, 133.9, 146.0, 160.3, 195.9. GC-MS (EI): *m/z* 248 (M^+, 100), 247 (M+-H, 35), 230 (4), 202 (6), 120 (9), 92(15). IR (KBr) ν (cm^{-1}): 711, 773, 892, 1070, 1118, 1215, 1265, 1298, 1385, 1434, 1468, 1560, 1604, 1705, 1886, 2851, 3063, 3422.

*Synthesis of 3-iodo-2-(methoxymethoxy)benzaldehyde (**S2**):* To a stirred solution of 2-hydroxy-3-iodobenzaldehyde (**S1**) (88.7 mmol, 22.0 g) in dry DMF (60 mL) were added K_2CO_3 (88.7 mmol, 12.2 g) and MOMCl (88.7 mmol, 6.9 mL) under a N_2 atmosphere. The color change from yellowish brown to yellow instantaneously occurs. After 16 h at room temperature the mixture was diluted with water (150 mL), extracted with EtOAc, and washed with brine. The organic phase was dried over Na_2SO_4 and concentrated under vacuum. The crude product was purified by distillation using Kuegelrohr apparatus affording the final product as a yellow oil (81 %). ^{1}H-NMR (300 MHz, $CDCl_3$) δ = 10.26 (s, 1H), 8.03 (dd, *J* = 1.5, 7.8 Hz, 1H), 7.80 (dd, *J* = 1.5, 7.8 Hz, 1H), 7.00 (t, *J* = 7.8 Hz, 1H), 5.14 (s, 2H), 3.59 (s, 3H). $^{13}C\{^{1}H\}$-NMR (75.5 MHz, $CDCl_3$) δ = 190.1, 160.1, 146.3, 145.5, 128.9, 126.8, 101.6, 93.5, 58.6. GC-MS (EI): *m/z* 292 (M^+, 35), 277 (M^+-CH_3, 5), 247 (M^+-C_2H_5O, 30), 246 (35), 92 (20), 63(15), 45(100). IR (NaCl) ν (cm^{-1}): 754, 844, 923, 984, 1108, 1159, 1228, 1244, 1312, 1400, 1429, 1521, 1558, 1616, 1695, 1844, 1897, 1982, 2075, 2340, 2358, 2739, 2829, 2951, 3006, 3060, 3337,

*Synthesis of tris(3-iodo-2-(methoxymethoxy)benzyl)amine (**S3**):* To a stirred solution of 3-iodo-2-(methoxymethoxy)benzaldehyde (**S2**) (58.2 mmol, 17.0 g) in dry THF (60 mL) was added ammonium acetate (18.4 mmol, 1.42 g) under a N_2 atmosphere. The mixture was stirred at room temperature for 1 h. Then, sodium triacetoxy borohydride (83.2 mmol, 17.6 g) was added and the suspension was stirred at room temperature. The reaction was monitored by TLC (petroleum ether/AcOEt 7:3). After 18 h the mixture was evaporated to dryness, dissolved EtOAc (100 mL), washed with sat. $NaHCO_3$ aq. (100 mL), and brine. The organic phase was dried over Na_2SO_4 and concentrated under vacuum. The crude product was purified by crystallization in CH_3CN affording the final product as a white solid (70%).

^{1}H-NMR (300 MHz, $CDCl_3$) δ = 7.66 (dd, *J* = 1.6, 7.9 Hz, 3H), 7.62 (dd, *J* = 1.6, 7.9 Hz, 3H), 6.86 (t, *J* = 7.9 Hz, 3H), 4.97 (s, 6H), 3.72 (s, 6H), 3.57 (s, 9H). ^{13}C{^{1}H}-NMR (75.5 MHz, $CDCl_3$) δ = 157.2, 139.7, 136.0, 131.4, 127.9, 101.8, 94.0, 59.5, 54.3. MS (ESI): *m/z* calc. 845.24; exp. 846.0 [M + H]$^+$. IR (KBr) ν (cm^{-1}): 733, 770, 797, 945, 1079, 1160, 1290, 1363, 1397, 1444, 1466, 1580, 2340, 2359, 2824, 2887, 2928, 2949, 2979, 3065.

*Synthesis of tris-(2-(methoxymethoxy)-3-(3'-formylbenzyl)-benzyl)amine (**S4**): Tris*-(3-iodo-2-(methoxymethoxy)benzyl)amine (**S3**) (4.7 mmol, 4.0 g) and (3-formylphenyl)boronic acid (28.4 mmol, 4.3 g) were added in a Schlenk tube under nitrogen. Degassed 1,4-Dioxane (70 mL), degassed K_2CO_3 aq. (2 M, 70 mL), and $Pd(PPh_3)_4$ (0.47 mmol, 546 mg) were added and the reaction was stirred at 100 °C. The reaction was monitored TLC (CH_2Cl_2). After 15 h, the reaction mixture was cooled down to room temperature, filtered over Celite and then concentrated under vacuum. The residue was diluted with CH_2Cl_2 (50 mL), washed with $NaOH_{aq}$ 1M (2 x 50 mL) and $NaCl_{aq}$ sat. (2 x 50 mL). The organic phase was dried over anhydrous Na_2SO_4, concentrated under reduced pressure and the residue was purified by column chromatography on silica gel (petroleum ether/ethyl acetate 6:4), affording a white powder (86%). ^{1}H-NMR (300 MHz, $CDCl_3$) δ = 10.1 (s, 3H), 8.1 (t, *J* = 1.5 Hz, 3H), 7.88 (m, 9H), 7.62 (t, *J* = 7.6 Hz, 3H), 7.30 (dd, *J* = 1.5, 4.6 Hz, 6 H), 4.59 (s, 6H), 3.94 (s, 6H), 3.14 (s, 9H). ^{13}C{^{1}H}-NMR (75.5 MHz, $CDCl_3$) δ = 184.6, 153.1, 140.0, 136.4, 135.4, 133.9, 133.5, 130.8, 129.8, 129.4, 128.9, 128.0, 124.7, 99.6, 57.9, 52.6. MS (ESI): *m/z* calc. 779.31; exp. 780.6 [M + H]$^+$. IR (KBr) ν (cm^{-1}): 732, 787, 960, 1067, 1180, 1304, 1401, 1449, 1576, 1600, 1699, 1734, 2724, 2825, 2934, 3058.

Synthesis of Tris-(2-hydroxy-3-(3'-formylbenzyl)-benzyl)amine (**1b**). *Tris*-(2-(methoxymethoxy)-3-(3'-formylbenzyl)-benzyl)amine **S4** (2.8 mmol, 2.5 g) was dissolved in THF (84 mL) and then HCl 1.25 M in methanol (16.8 mL) was added. The reaction mixture was heated at 50 °C for 18 h. A solution of HCl_{aq} 1M was added and the reaction was vigorously stirred for 3 h. Then $NaHCO_{3aq}$ sat. was added till pH = 8 was reached. The residue was extracted with EtOAc. The organic phases were dried over anhydrous Na_2SO_4 and concentrated under vacuum. The product, a brownish foam, was used without any further purification (92%). ^{1}H-NMR (300 MHz, $CDCl_3$) δ = 9.77 (s, 3H), 7.74 (s, 3H), 7.65 (d, *J* = 7.5 Hz, 3H), 7.54 (d, *J* = 7.5 Hz, 3H), 7.37 (t, *J* = 7.5 Hz, 3H), 7.00 (d, *J* = 7.5 Hz, 3H), 6.96 (d, *J* = 7.5 Hz, 3H), 6.74 (t, *J* = 7.5 Hz, 3H), 3.76 (s, 6H). ^{13}C{^{1}H}-NMR (75.5 MHz, $CDCl_3$) δ = 192.10, 152.06, 138.43, 136.23, 135.3, 130.8, 130.5, 130.1, 128.8, 128.1, 127.6, 123.9, 120.3, 57,9. MS (ESI): *m/z* calc. 647.23; exp. 648.5 [M + H]$^+$. IR (KBr) ν (cm^{-1}): 743, 772, 868, 907, 1071, 1185, 1262, 1303, 1383, 1425, 1462, 1576, 1595, 1696, 2730, 2828, 2962, 3372.

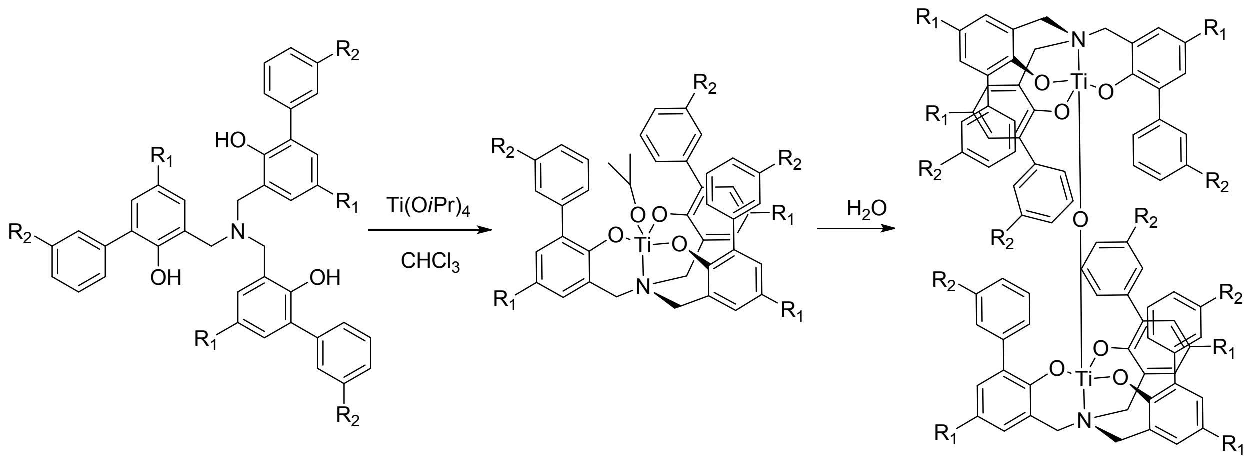

General synthesis of mononuclear titanatranes **3a**, **3b**. Complexes were prepared in glovebox by mixing homogeneous solutions of the corresponding ligands (0.7 mmol in 7 mL, 0.10 M) and Ti(O*i*-Pr)$_4$ (0.7 mmol in 3.5 mL, 0.2 M) in dry $CHCl_3$ and stirring the resulting solution overnight under inert atmosphere. Bright yellow/orange solutions were obtained which were used without removing the three equivalents of *i*-PrOH released from the metal precursor. In all cases resonances relative to free *iso*-propanol released in the reaction were present in the NMR spectra: ^{1}H-NMR (200 MHz, $CDCl_3$): 4.04 (1H, *hept*, J = 6.1 Hz), 1.22 (6H, *d*, J = 6.1 Hz). ^{13}C{^{1}H}-NMR: (50.03 MHz, $CDCl_3$) δ 64.5 (*C*H), 25.1 (*C*H$_3$).

3a: ^{1}H-NMR (300 MHz, $CDCl_3$) δ = 9.51 (*brs,* 3H), 7.58-7.28 (*m*, 21H), 4.44 (*brs*, 1H), 3.3 (*brs*, 6H), 0.83 (d, J = 6.0 Hz, 6H).

3b: ^{1}H-NMR (300 MHz, $CDCl_3$) δ = 9.94 (s, 3H), 7.98 (s, 3H), 7.95 (d, J = 7.6 Hz, 3H), 7.74 (d, J = 7.6 Hz, 3H), 7.49 (t, J = 7.6 Hz, 3H), 7.24 (d, J = 7.6 Hz, 3H), 7.10 (d, J = 7.6 Hz, 3H), 6.87 (t, J = 7.6 Hz, 3H), 4.43 (*hept*, J = 6.0 Hz, 1H), 3.69 (s, 6H), 0.68 (d, J = 6.0 Hz, 6H).

General synthesis of Ti(IV) TPA μ-oxo complexes **4a, 4b**. To the above *in situ* prepared mononuclear complex **3a, 3b** solutions water (0.25 ml for 0.7 mmol of complex) was added and the mixture was stirred at r.t. for 1-2 h. The suspension was concentrated under pressure and the yellow solid, precipitated with diethyl ether or *n*-hexane, was recovered by filtration or centrifugation (98 %).

4a: ^{1}H-NMR (300 MHz, $CDCl_3$) δ = 9.97 (*s*, 6H), 7.85 (*d*, J = 5 Hz, 12H), 6.66-6.57 (*m,* 24H), 6.31 (*t*, J = 12 Hz, 6H), 4.32 (*d*, J = 14 Hz, 6H), 3.51 (*d*, J = 14 Hz, 6H). ^{13}C{^{1}H}-NMR (50.03 MHz, DMSO-d_6) δ = 190.38, 165.15, 138.42, 137.56, 132.68, 130.25, 130.01, 128.96, 128.05, 127.50, 126.89, 126.66, 126.18, 63.18. MS (ESI): *m/z* calc. 1401.31; exp. 1423.4 [M + Na]$^+$, 1401.2 [M]$^-$, 1445.2 [M + HCOO]$^-$, 1513.1 [M + CF_3COO]$^-$. IR (KBr) ν (cm^{-1}): 750, 867, 973,

1000, 1031, 1110, 1168, 1281, 1384, 1419, 1441, 1464, 1521, 1593, 1635, 1684, 2336, 2361, 2843, 3053, 3420.

4b: ^{1}H-NMR: (300 MHz, $CDCl_3$) δ = 9.20 (*s*, 6H), 7.33-7.24 (*m*, 18H), 7.07 (*t*, *J* = 7.5 Hz, 6H), 6.95 (*d*, *J* = 7.5 Hz, 6H), 6.80 (*d*, *J* = 7.5 Hz, 6H), 6.73 (*t*, *J* = 7.5 Hz, 6H), 4.40 (*d*, *J* = 14.2 Hz, 6H), 3.31 (*d*, *J* = 14.3 Hz, 6H). ^{13}C{^{1}H}-NMR: (75.5 MHz, $CDCl_3$) δ 191.97, 159.30, 138.77, 136.04, 131.52, 129.92, 129.78, 128.49, 126.77, 125.33, 122.02, 59.07. MS (ESI): *m/z* calc. 1401.31; exp. 1423.4 $[M + Na]^+$, 1401.2 $[M]^-$, 1445.2 $[M + HCOO]^-$, 1513.1 $[M + CF_3COO]^-$. IR (KBr) ν (cm^{-1}): 693, 752, 802, 891, 1163, 1193, 1240, 1270, 1366, 1378, 1453, 1588, 1696, 2847, 3059, 3439.

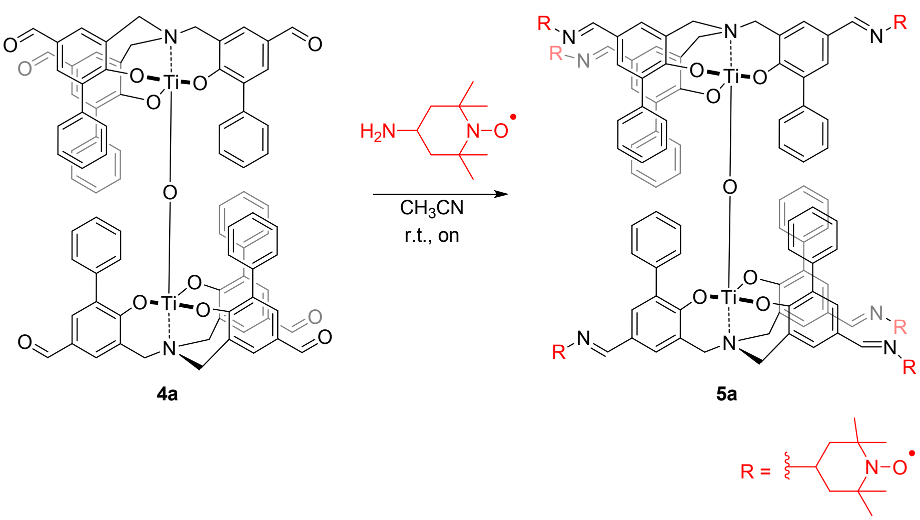

Synthesis of compound **5a**. µ-oxo Ti complex **4a** (50 mg, 0.04 mmol) and free radical 4-amino-TEMPO (49 mg, 0.29 mmol) were dissolved in CH_2Cl_2 (4 mL). The mixture was stirred overnight. Hexane was added to the solution until the formation of a yellow precipitate. The mixture was stirred for 10 min and filtered. The solid was washed with CH_2Cl_2 −hexane (1:10) several times and dried to afford 71 mg of the imine **5a** (86%) as a yellow solid. IR (KBr): 2973, 2931, 2857, 1637, 1598, 1467, 1346, 1242, 1171 cm^{-1}. Anal. Calcd. ($C_{138}H_{162}N_{14}O_{13}Ti_2$): C, 71.43; H, 7.04; N, 8.45. Found: 70.88; H, 7.08; N, 8.65.

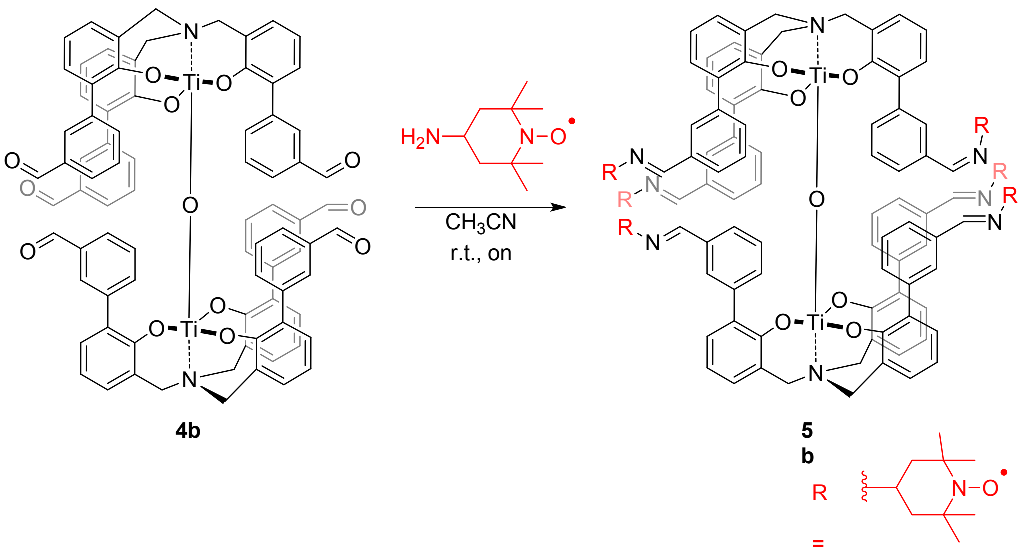

Synthesis of compound **5b**. μ-oxo Ti complex **4b** (50 mg, 0.04 mmol) and free radical 4-amino-TEMPO (49 mg, 0.29 mmol) were dissolved in CH_3CN (4 mL). The mixture was stirred overnight. Hexane was added to the solution until the formation of a yellow precipitate. The mixture was stirred for 10 min and filtered. The solid was washed with CH_3CN −hexane (1:10) several times and dried to afford 79 mg of the imine **5b** (95%) as a yellow solid. IR (KBr): 2973, 2932, 2860, 1643, 1588, 1455, 1266, 1241, 1175 cm^{-1}. Anal. Calcd. ($C_{138}H_{162}N_{14}O_{13}Ti_2$): C, 71.43; H, 7.04; N, 8.45. Found: 71.08; H, 7.12; N, 8.55.

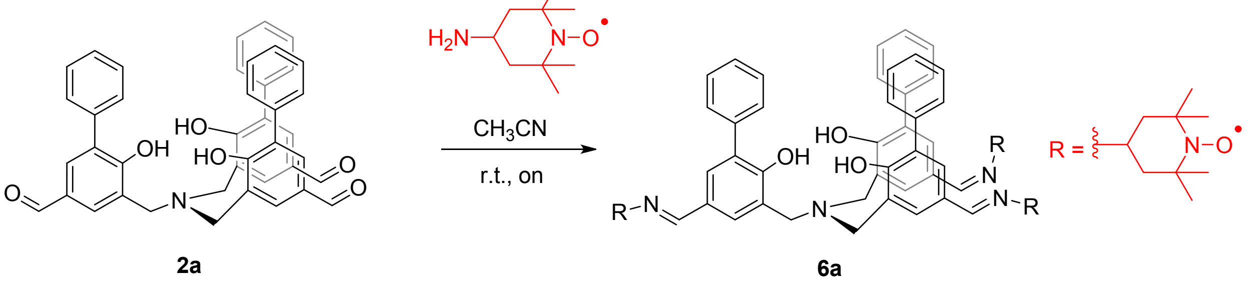

Synthesis of compound **6a**. Ligand **2a** (50 mg, 0.08 mmol) and free radical 4-amino-TEMPO **2** (53 mg, 0.31 mmol) were dissolved in CH_3CN (2 mL). The mixture was stirred overnight. Hexane was added to the solution until the formation of an orange precipitate. The mixture was stirred for 10 min and filtered. The solid was washed with CH_3CN −hexane (1:10) several times and dried to afford 82mg of the imine **6a** (96%) as an orange solid. IR (KBr): 2973, 2933, 2863, 1640, 1600,

1468, 1362, 1243, 1173 cm^{-1}. MS (ESI) calcd for $C_{69}H_{84}N_7O_6$ $(M+H)^+$ 1107.8, found 1107.8. Anal. Calcd. ($C_{69}H_{84}N_7O_6$): C, 74.83; H, 7.65; N, 8.85. Found: C, 74.95; H, 7.55; N, 8.75.

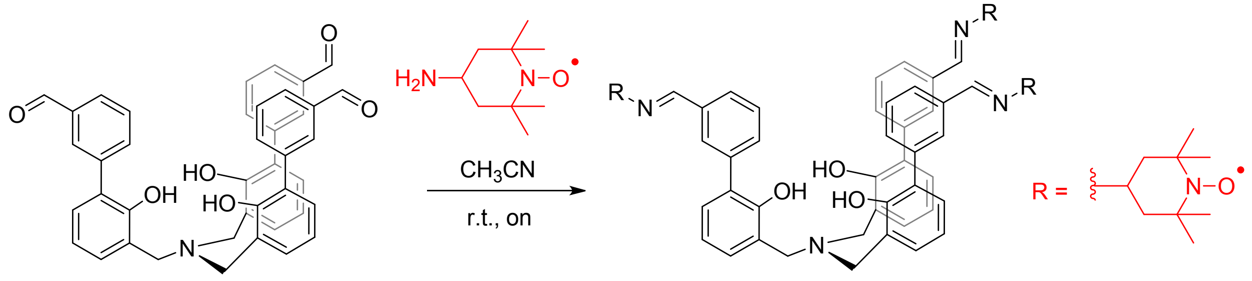

2b 6b

Synthesis of compound 6b. Ligand **2** (50 mg, 0.08 mmol) and free radical 4-amino-TEMPO (53 mg, 0.31 mmol) were dissolved in CH_3CN (2 mL). The mixture was stirred overnight. Hexane was added to the solution until the formation of an orange precipitate. The mixture was stirred for 10 min and filtered. The solid was washed with CH_3CN −hexane (1:10) several times and dried to afford 79 mg of the imine **6b** (93%) as a pink solid. IR (KBr): 2973, 2932, 2863, 1642, 1591, 1468, 1381, 1243, 1176 cm^{-1}. MS (ESI) calcd for $C_{69}H_{84}N_7O_6$ $(M+H)^+$ 1107.8, found 1107.8. Anal. Calcd. ($C_{69}H_{84}N_7O_6$): C, 74.83; H, 7.65; N, 8.85. Found: C, 74.35; H, 7.75; N, 8.92.

3. NMR spectra

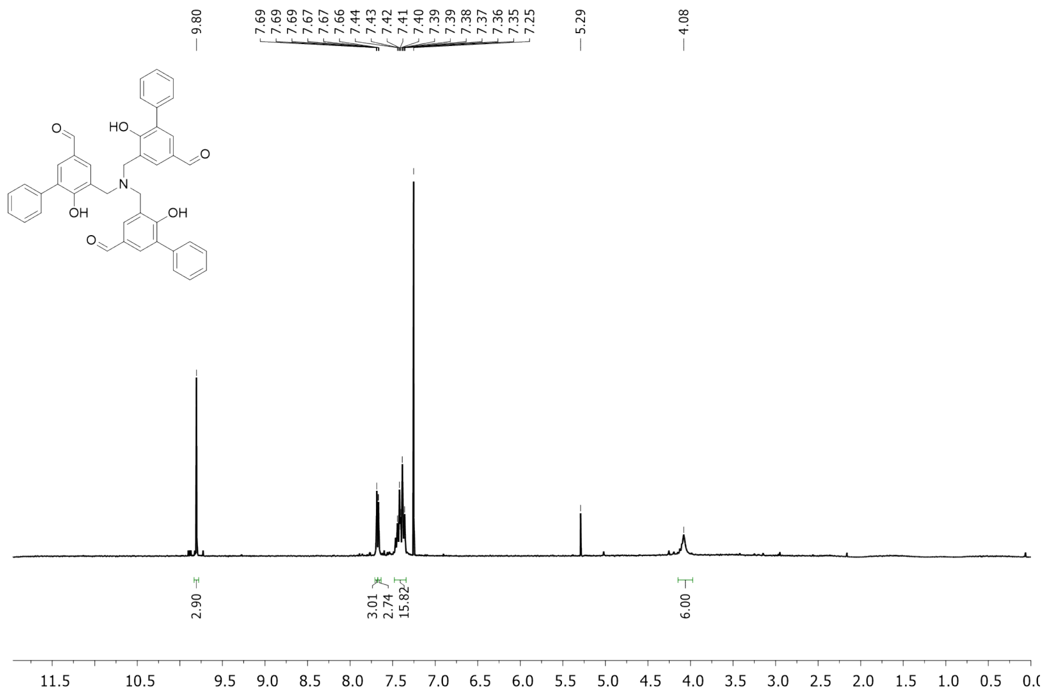

Figure S1. ^{1}H-NMR of **1a** in $CDCl_3$.

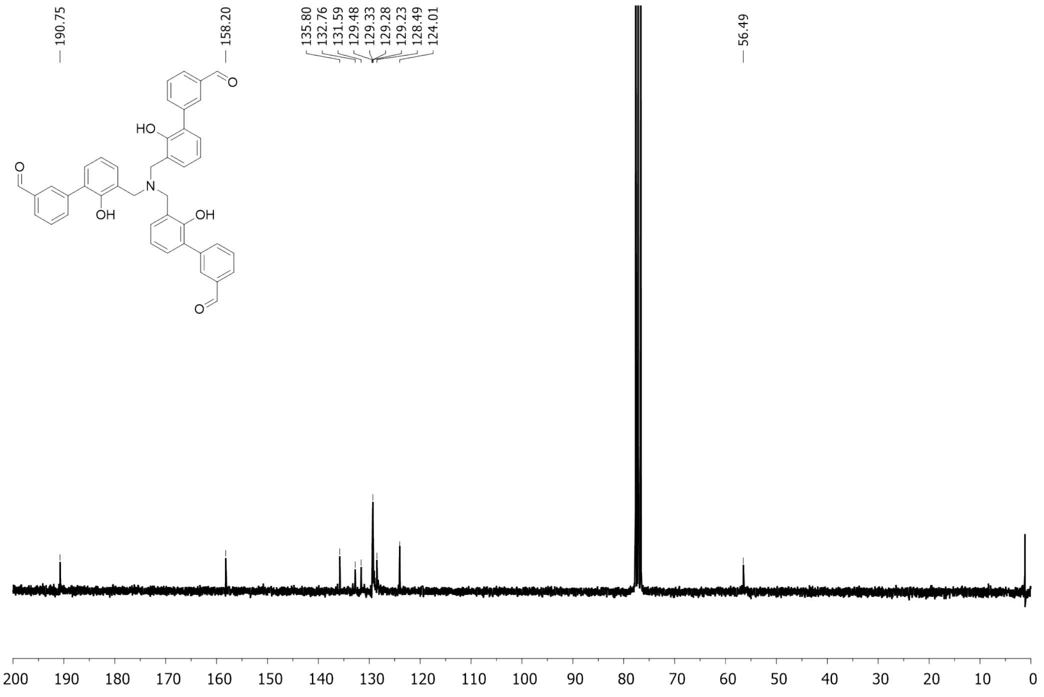

Figure S2. $^{13}C\{^{1}H\}$-NMR of **1a** in $CDCl_3$.

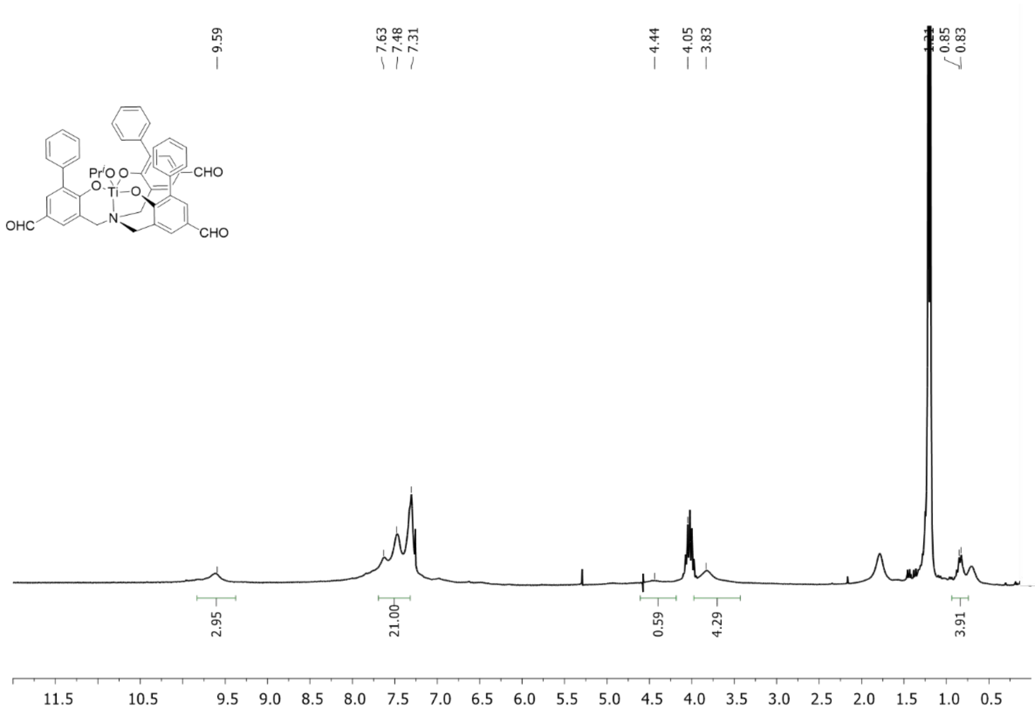

Figure S3. ^{1}H-NMR of **3a** in $CDCl_3$.

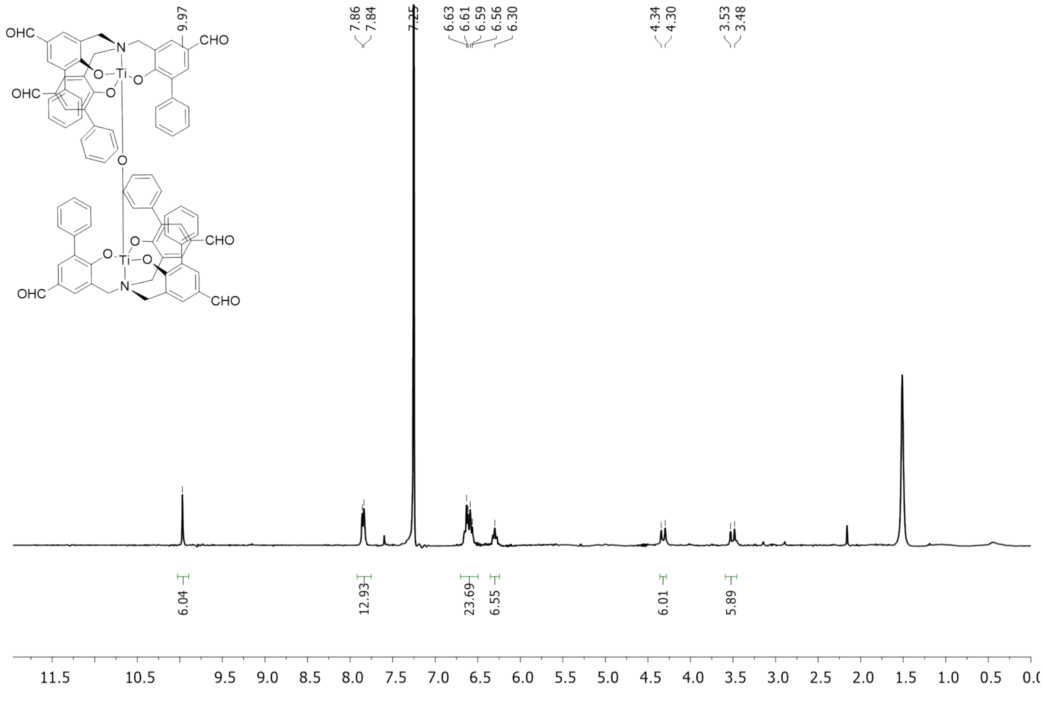

Figure S4. ^{1}H-NMR of **4a** in $CDCl_3$.

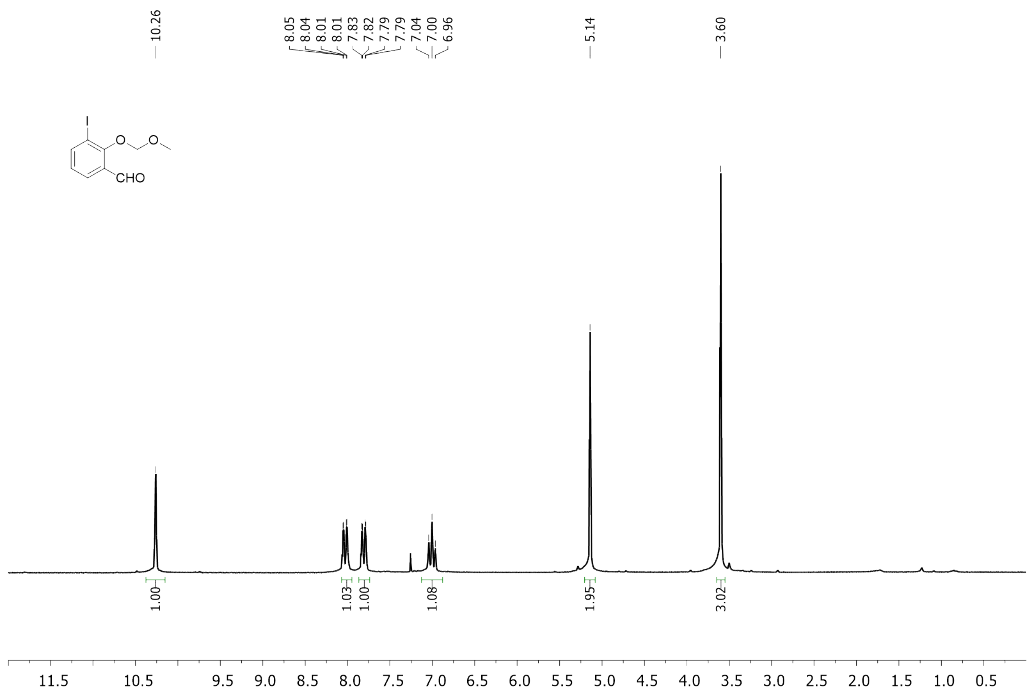

Figure S5. ^{1}H-NMR of **S2** in $CDCl_3$.

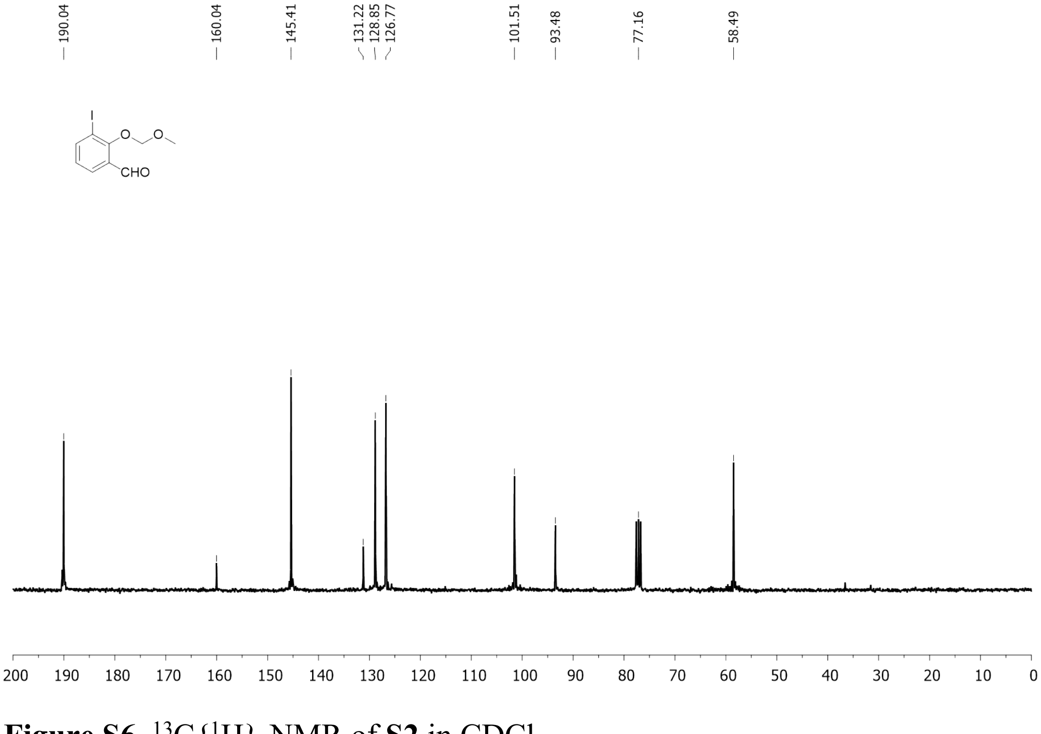

Figure S6. $^{13}C\{^{1}H\}$-NMR of **S2** in $CDCl_3$.

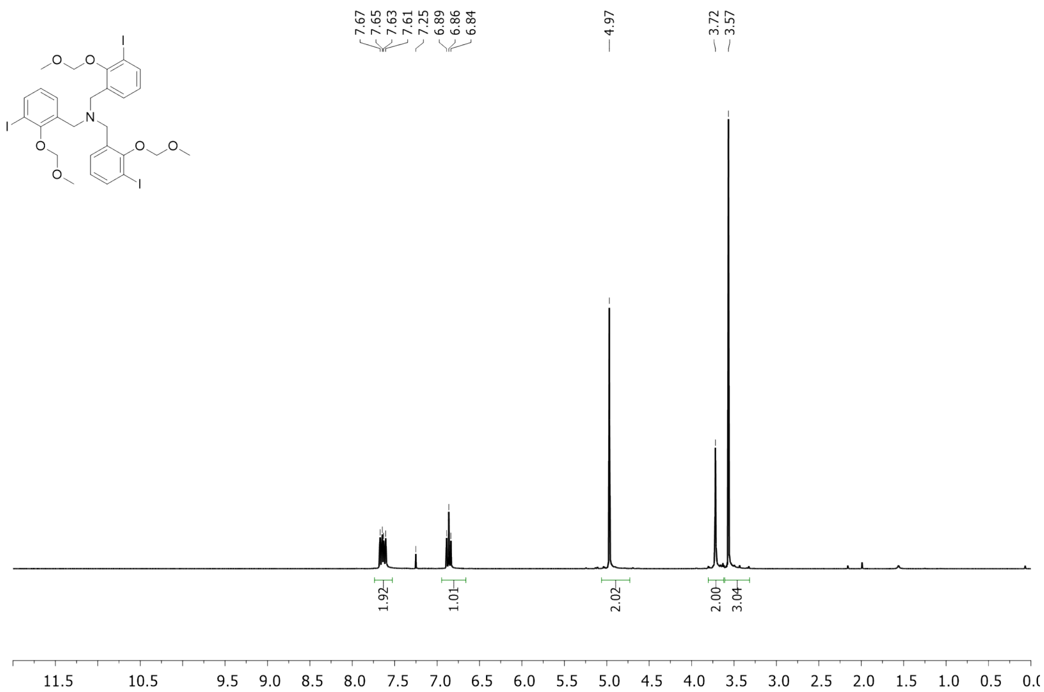

Figure S7. ^{1}H-NMR of **S3** in $CDCl_3$.

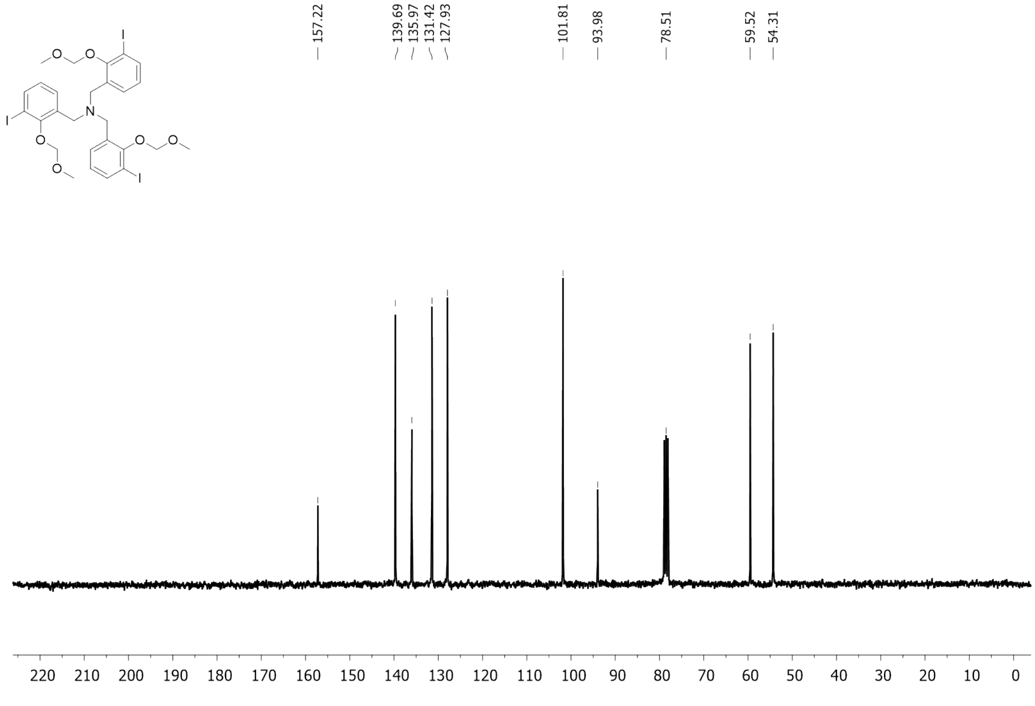

Figure S8. $^{13}C\{^{1}H\}$-NMR of **S3** in $CDCl_3$.

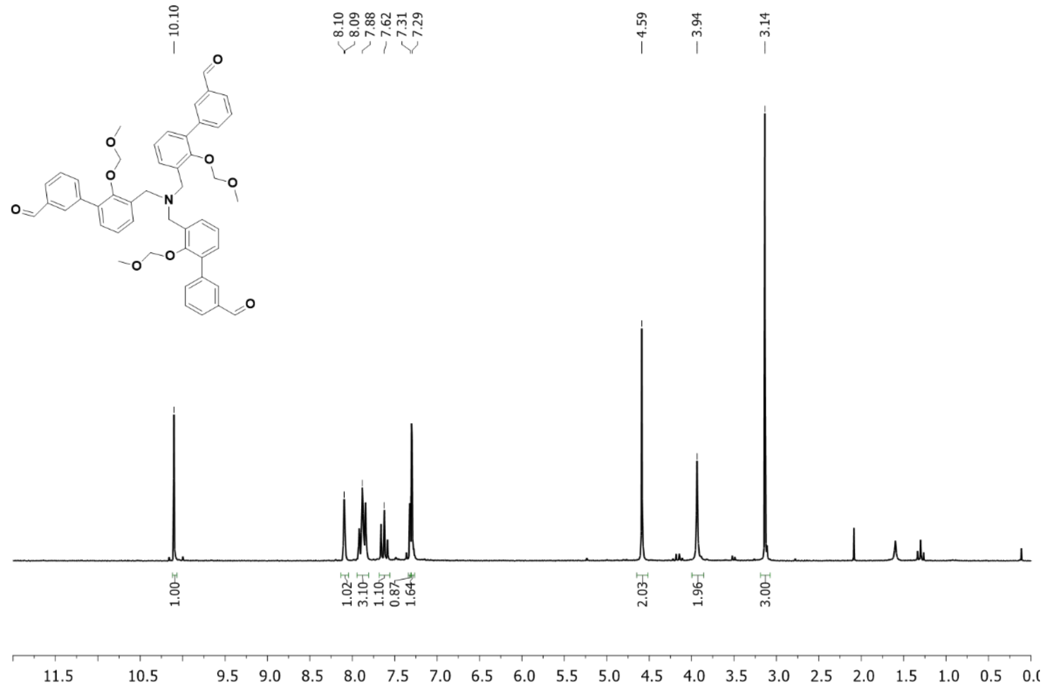

Figure S9. ^{1}H-NMR of **S4** in $CDCl_3$.

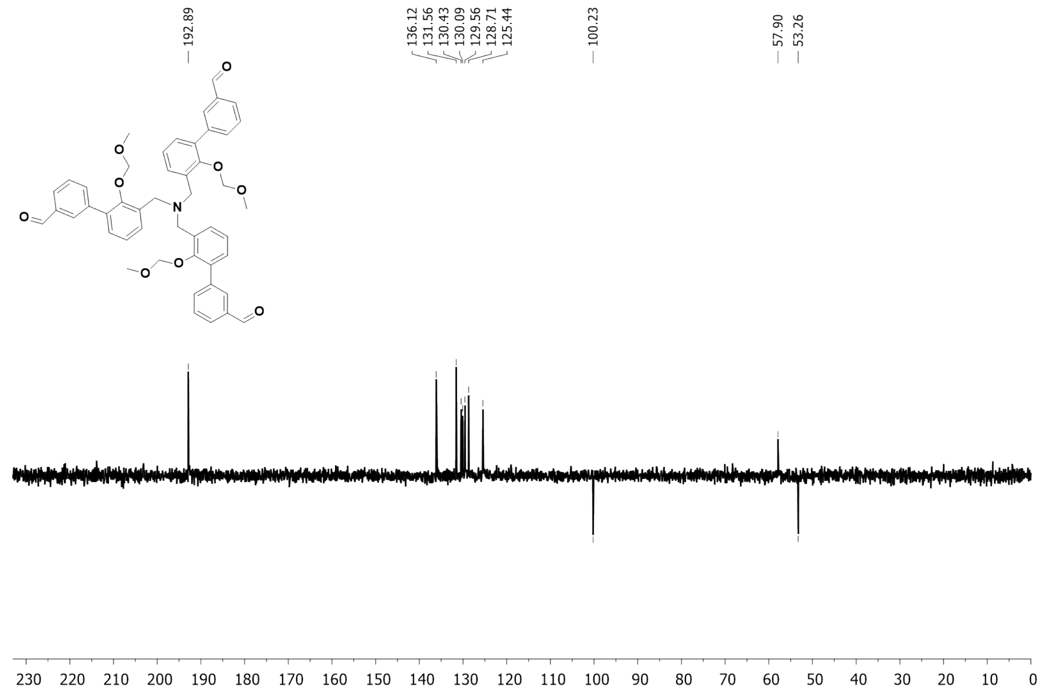

Figure S10. DEPT135-NMR of **S4** in $CDCl_3$.

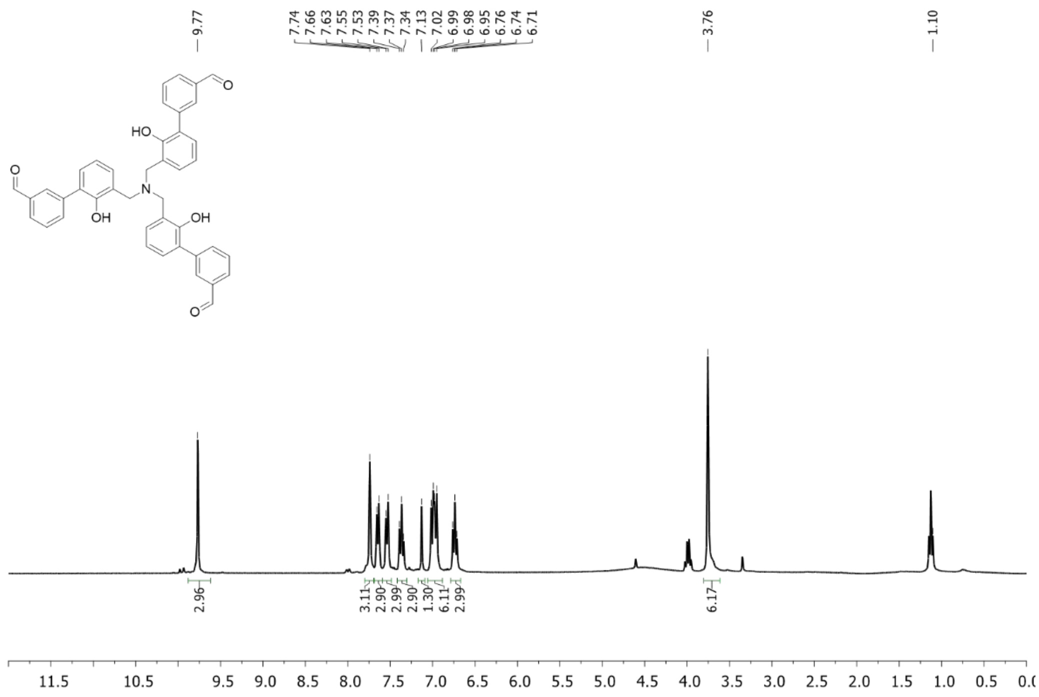

Figure S11. ^{1}H-NMR of **2b** in $CDCl_3$.

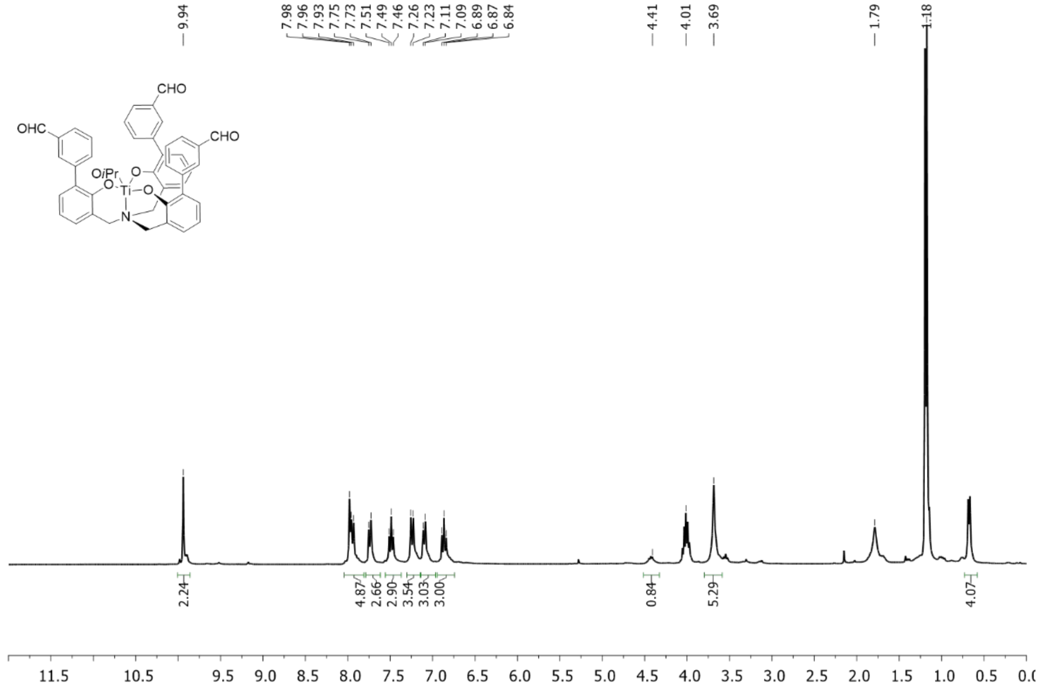

Figure S12. ^{1}H-NMR of **3b** in $CDCl_3$.

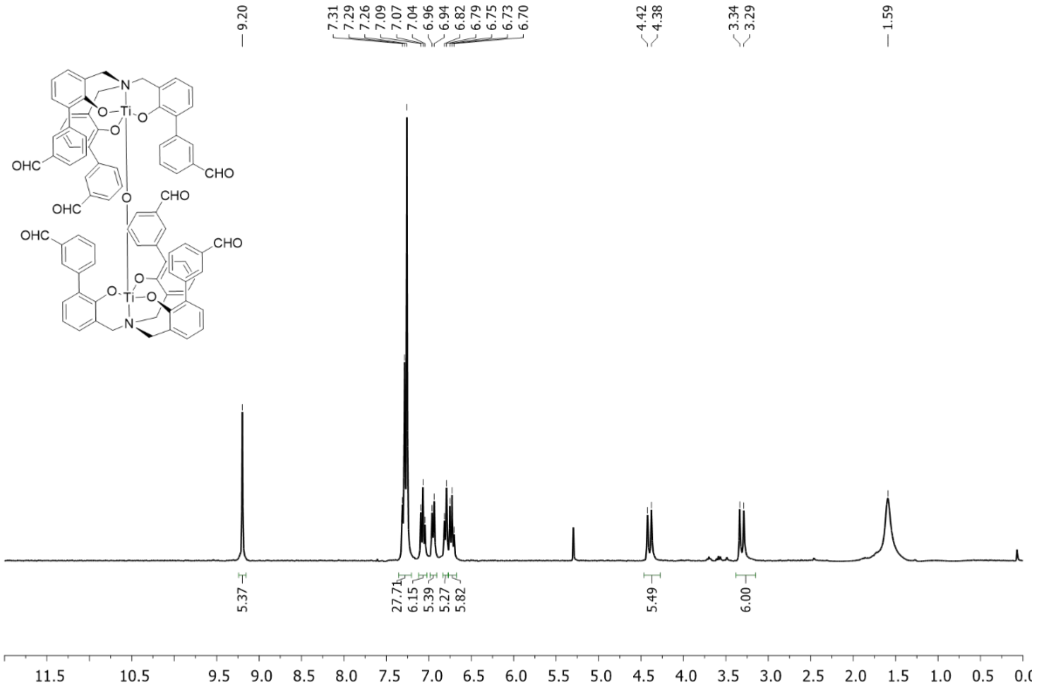

Figure S13. ^{1}H-NMR of **4b** in $CDCl_3$.

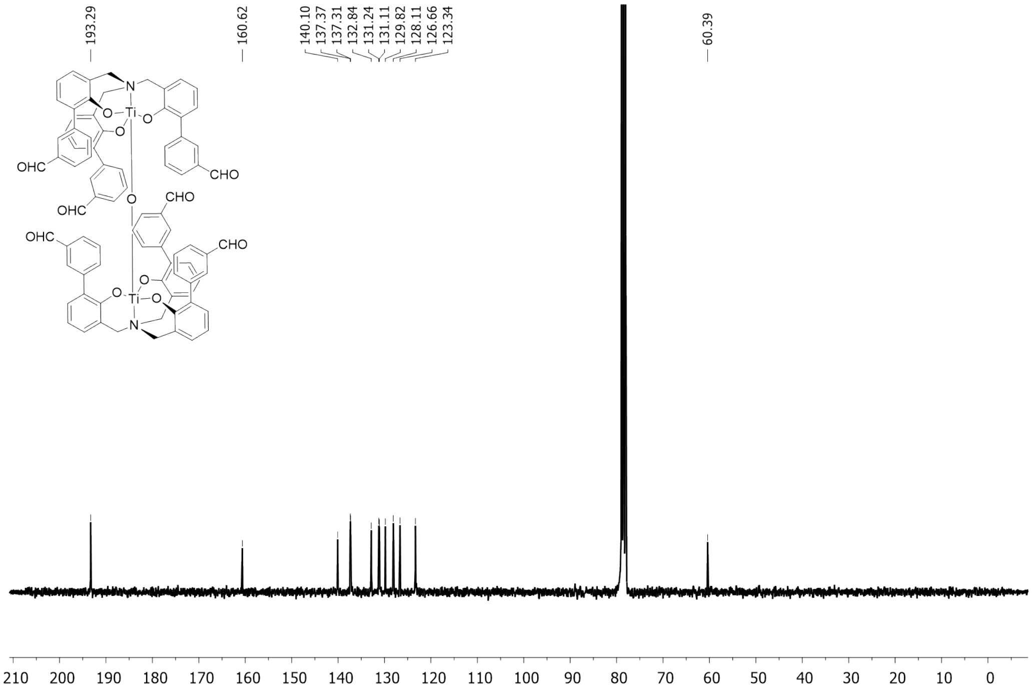

Figure S14. $^{13}C\{^{1}H\}$-NMR of **4b** in $CDCl_3$.

4. Quantitative EPR study

The full functionalization with radicals of all compounds was quantitatively determined by EPR spectroscopy. The intensity (double integral) of the EPR signal of all compounds was measured, in the same conditions. TEMPO-derived compounds presented an experimental number of radicals very close to the theoretical value and in agreement within the ±10% tolerance of EPR measurements, showing the complete functionalization of all compounds (see Table S1).

Table S1. Normalized EPR signal double integral of ligands **6a-6b** and μ-oxo complexes **5a-5b** at 130 K in DCM:TOL 1:1.

Compound	nº of theor. TEMPO units	Conc.	Normalized EPR double integral	nº of exp. TEMPO units
**6a**	3	1mM	3089	3.0
**6b**	3	1mM	2963	2.9
**5a**	6	1mM	6112	6.0
**5b**	6	1mM	5916	5.8

5. Frozen solution EPR spectra of compounds 6a-6b

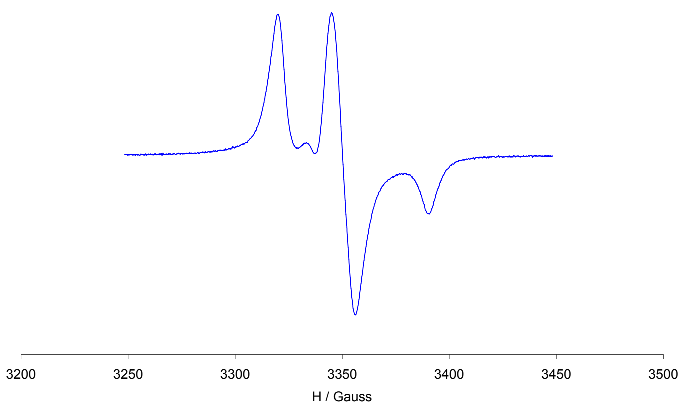

Figure S15. EPR spectrum of compound **6a** in dichloromethane:toluene 1:1 at 130 K.

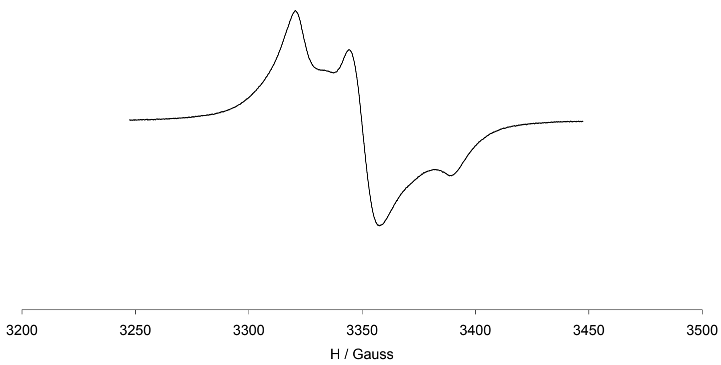

Figure S16. EPR spectrum of compound **6b** in dichloromethane:toluene 1:1 at 130 K.

6. Half-field EPR spectra of ligands 6a-6b

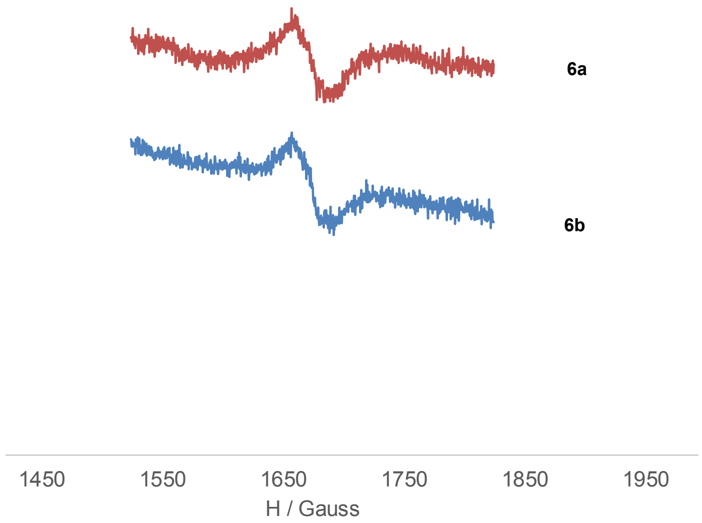

Figure S17. $|\Delta m_s|= 2$ transition at half-field spectra of ligands **6a-6b** in DCM:TOL 1:1 at 130 K.

7. EPR study of monoradical TEMPO

TEMPO free radical was studied at different concentrations (1, 3, 6 and 12 mM). As expected, it did not present half-field transition band. Only in the most concentrated solutions (6 and 12 mM) a little band was observed but, in this case, due to intermolecular interactions favoured at very high concentration (see Table S2). This band was much smaller than those in the polyradical species because of the lack of intramolecular radical interactions. The same effect was also reflected in the d_1/d ratio, which slightly increased from 0.51 to 0.54 with the increase of concentration due to the closer distance between molecules (Table S2).

8. Cyclic voltammetries of 6a-6b, 5a-5b and TEMPO

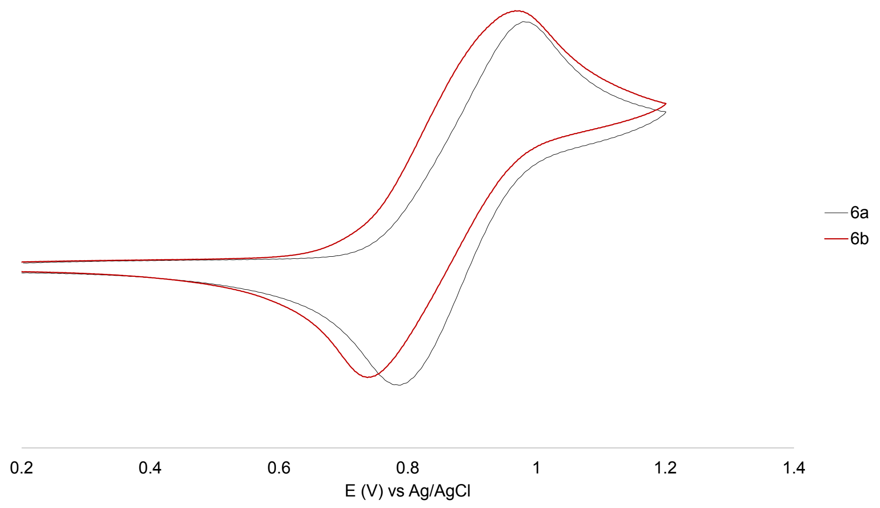

Figure S18. Cyclic voltammetry of ligands **6a-6b** in DMF at a scan rate of 0.2 V/s, vs Ag/AgCl.

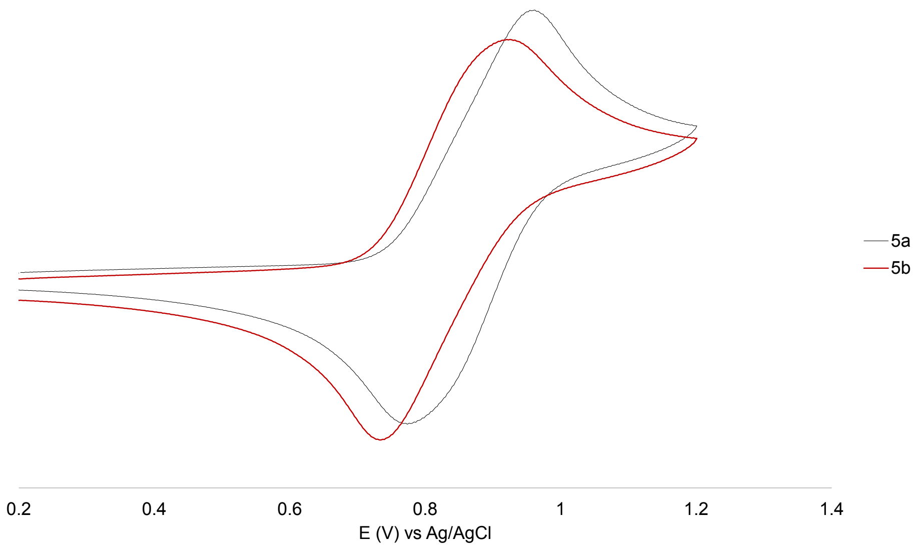

Figure S19. Cyclic voltammetry of μ-oxo complexes **5a-5b** in DMF at a scan rate of 0.2 V/s, vs Ag/AgCl.

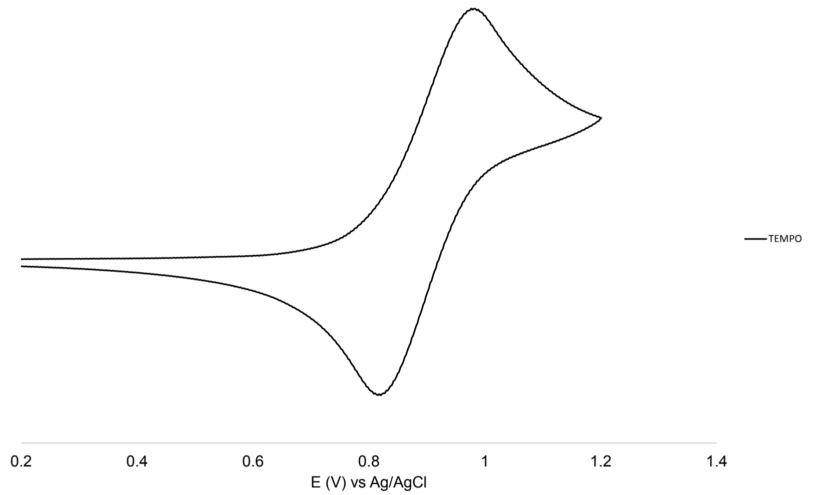

Figure S20. Cyclic voltammetry of TEMPO in DMF at a scan rate of 0.2 V/s, vs Ag/AgCl.

A)

B)

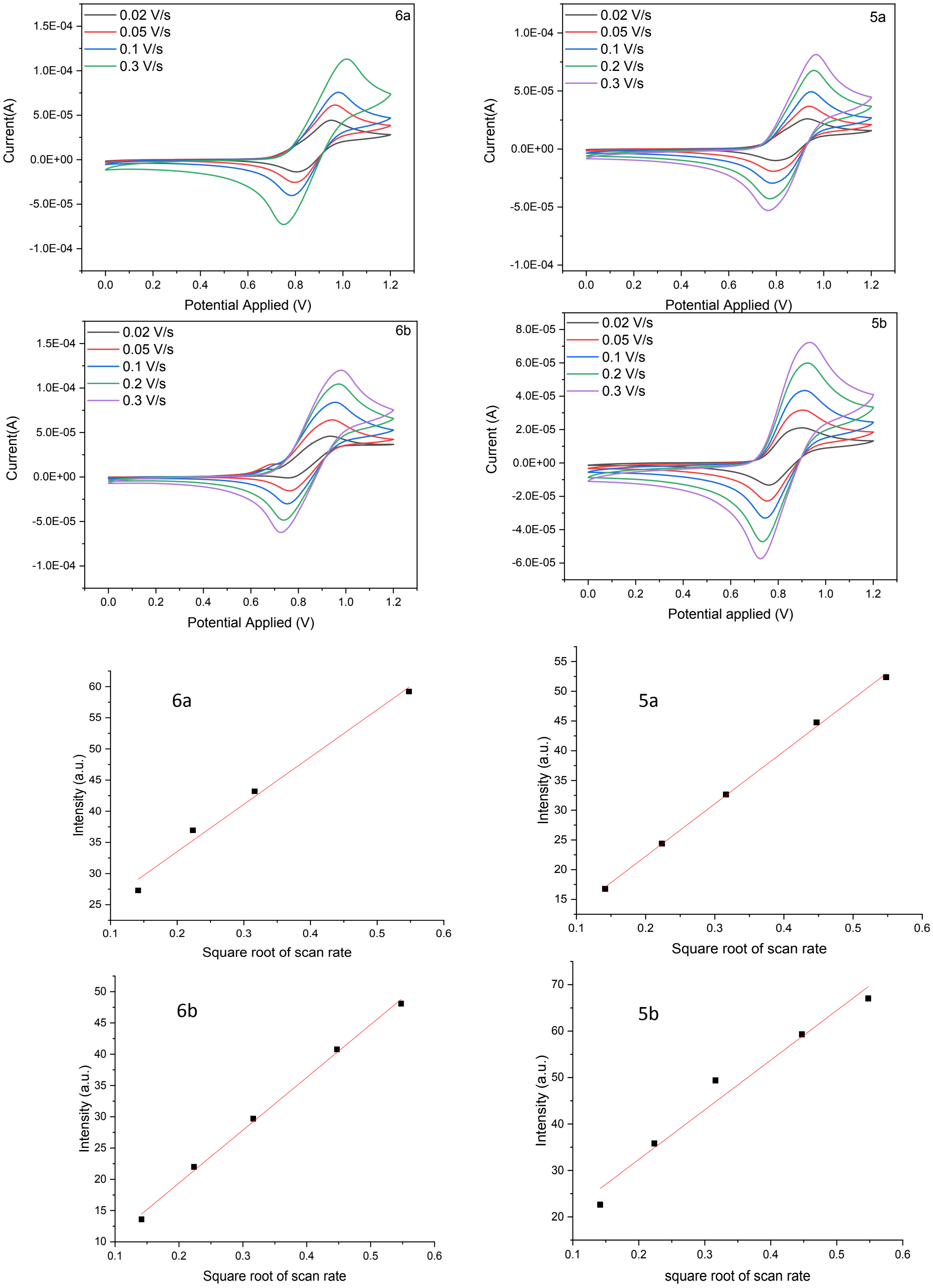

Figure S21. A) Cyclic voltammograms of polynitroxides **6a-6b** and **5a-5b** at different scan rates and B) the corresponding plots of the current intensity vs the square root of scan rate for them. The current intensity is proportional to the square root of the scan rate in all cases, indicating a reversible electron transfer reaction in terms of the diffusion layer thickness.

9. Charge and discharge galvanostatic pulses

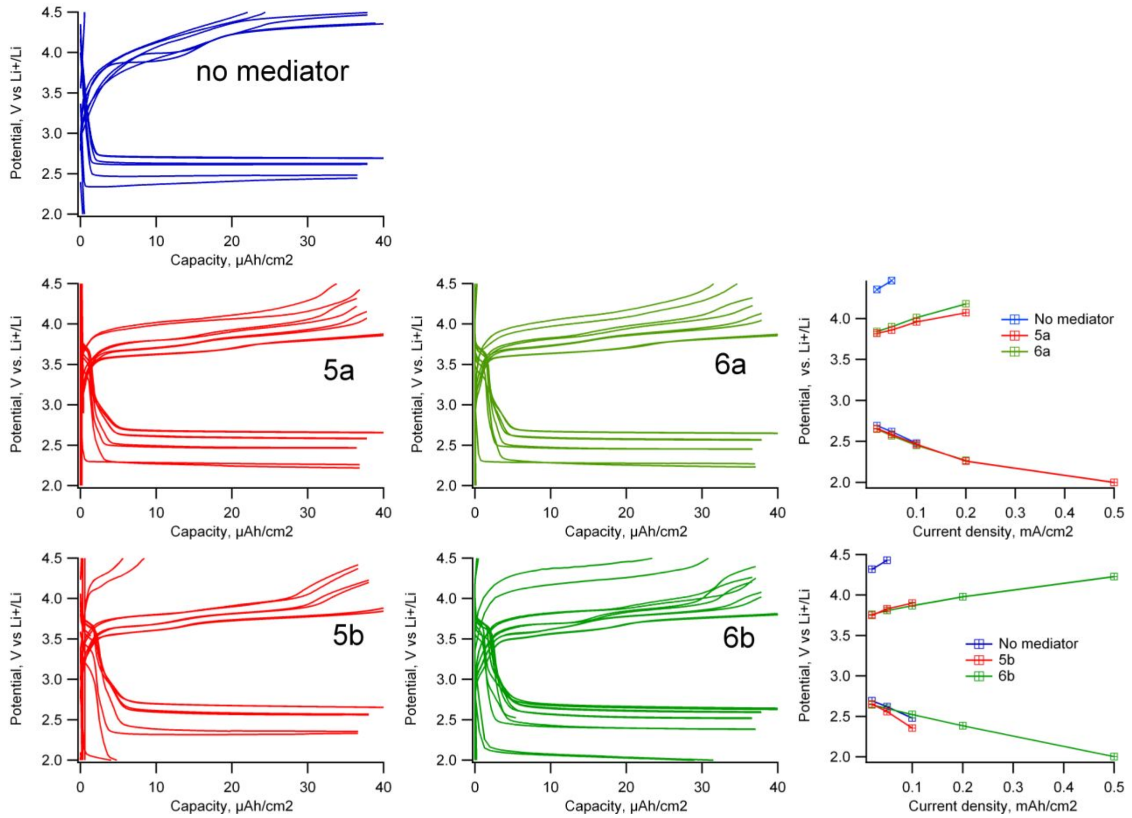

Figure S22. Charge and discharge galvanostatic pulses using different mediators in the electrolyte using current densities between 0.02 and 0.5 mA/cm^2 (left and middle column). Polarization curves plotted using plateaus values from galvanostatic pulses (right column).

a)

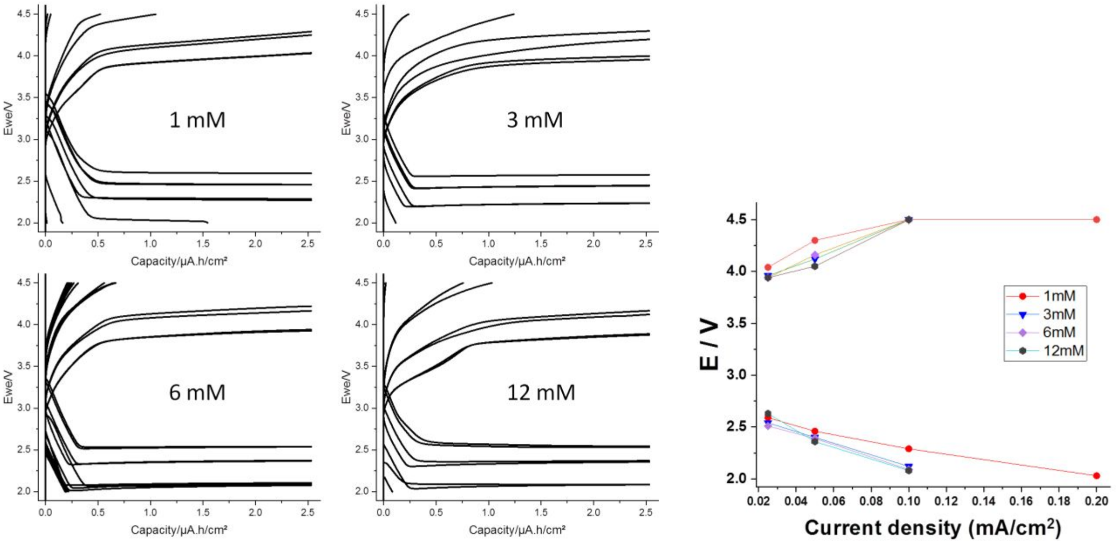

b)

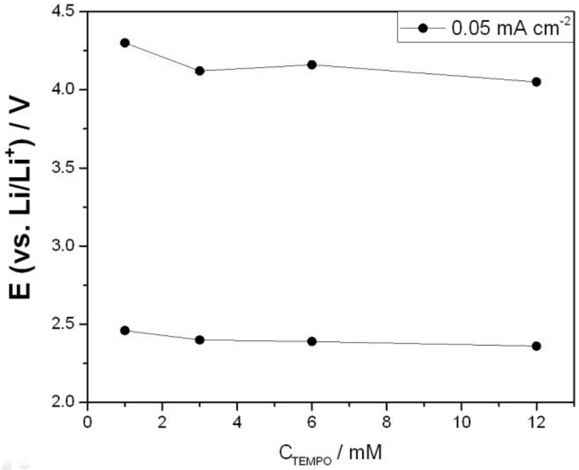

c)

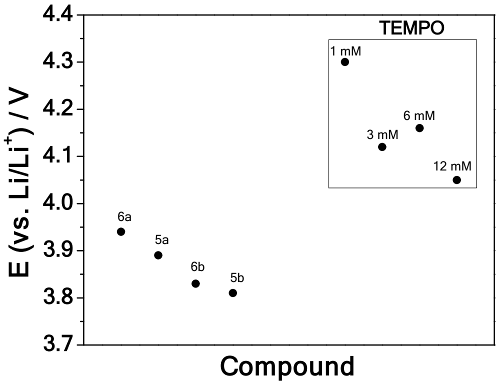

Figure S23. a) Charge and discharge galvanostatic pulses using for TEMPO free radical at different concentrations and current densities (left and middle column). Polarization curves plotted using plateaus values from galvanostatic pulses (right column). b) Charge and discharge plateau voltage (vs Li^+/Li) plotted versus TEMPO free radical concentration at 0.05 mA/cm^2. c) Comparison of charge plateau voltages for the different compounds at 0.05 mA/cm^2.

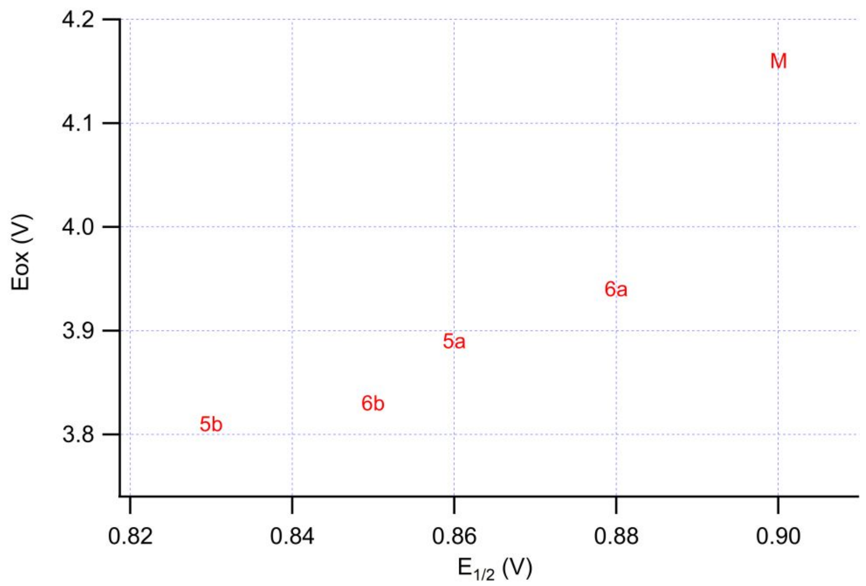

Figure S24. Relationship between charging potential (E_{ox}) and half wave potential ($E_{1/2}$). Compounds are identified with the code used along this report, while the letter "M" refers to the TEMPO monomer. Values are given for a constant TEMPO unit concentration of 6 mM (i.e. 1 mM complexes, 2 mM ligands and 6 mM monomer).

Table S2. Data of d_1/d ratio, half-field intensity values, E½ vs Ag/AgCl from the cyclic voltammetry and the voltage vs Li^+/Li at 0.05 mA/cm^2 for TEMPO free radical at 1, 3, 6 and 12 mM:

Conc.	d_1/d	$\|\Delta m_s\|$= 2 transition intensity	$E_{1/2}$ (V) vs Ag/AgCl	Voltage (V) *vs* Li^+/Li at 0.05 mA/cm^2
1 mM	0.51	-	0.88	4.3
3 mM	0.51	-	0.89	4.12
6 mM	0.52	0.38	0.90	4.16
12 mM	0.54	0.81	0.91	4.05

Table S3. Discharge and charge voltage vs. Li^+/Li at different current density for TEMPO free radical at 1, 3, 6 and 12 mM:

I (mA/cm^2)	1mM		3mM		6mM		12mM	
0.1	2.29	4.5	2.12	4.5	2.09	4.5	--	--
0.05	2.46	4.3	2.4	4.12	2.39	4.16	2.36	4.05
0.025	2.59	4.04	2.54	3.96	2.51	3.94	2.63	3.94

10. X-ray crystallography

Single crystal X-ray data for **5b** were collected at 123 K with Agilent Super-Nova dual source wavelength diffractometer with an Atlas CCD detector using multilayer optics monochromatized Mo- *K*α (λ = 1.54184 Å) radiation. The data collection and reduction were performed using the program *CrysAlisPro*,[3] the intensities are corrected for absorption with empirical method. The structure was solved with *SHELXT*[4] and refined by full-matrix least squares on F^2 using the *OLEX2,*[5] which utilizes the *SHELXL*-2015 module.[6] Anisotropic displacement parameters were assigned to non-H atoms. All the hydrogen atoms were refined using riding models with *U*eq(H) of 1.5*U*eq(parent) for terminal methyl groups, and 1.2 *U*eq(parent) for other groups. One of the TEMPO groups in the asymmetric unit is disordered over three positions. They were split according to the difference Fourier maps, and their occupancies were first freely refined and then fixed to 0.40, 0.32 and 0.28, respectively. Geometric restraints (DFIX and SADI), as well as the ADP restraints (SIMU) were used to treat the disordered groups. For the disordered atom attached with the imine-N were very close, for whom, EADP command was utilized to force them with the same thermal displacement parameters. Badly disordered solvent molecules were found in the lattice and were treated using the "SQUEEZE" protocol within PLATON.[7]

Crystal Data for 5b

$C_{138}H_{162}N_{14}O_{13}Ti_2$, *Mr* = 2320.63gmol^{-1}, Crystal dimensions: 0.09 x 0.18 x 0.23 mm, trigonal, space group *R*-3, *a* = 25.0202(7) Å, *c* = 34.6615(10) Å, *V* = 18791.3(12) Å^3, *Z* = 6, D_c = 1.230 Mg/m^3, μ= 0.195 mm^{-1}, F000 = 7416, *T* = 123.01(10) K, θ range for cell measurement: 1.97-25.25°, R_1 = 0.0729 (0.1233), *wR* = 0.1781 (0.2165), R_{int} = 0.0278, 13240 reflections are measured with 7565 independent reflections of which 4599 are I_o>2σ(I_o), 668 parameters, 572 restraints, *GooF* = 1.038, -0.413<Δρ<0.453 e/Å^3. CCDC 1986124 contains the supplementary data for this structure.

11.References

(1) Still, W. C.; Kahn, M.; Mitra, A. Rapid Chromatographic Technique for Preparative Separations with Moderate Resolution. *J. Org. Chem.* **1978**, *43* (14), 2923–2925. https://doi.org/10.1021/jo00408a041.

(2) Bender, C. L.; Hartmann, P.; Vračar, M.; Adelhelm, P.; Janek, J. On the Thermodynamics, the Role of the Carbon Cathode, and the Cycle Life of the Sodium Superoxide (NaO2) Battery. *Adv. Energy Mater.* **2014**, *4* (12), 1301863. https://doi.org/10.1002/aenm.201301863.

(3) CrysAlisPro 2012, Agilent Technologies. Version 1.171.36.35.

(4) Sheldrick, G. M. {\it SHELXT} {--} Integrated Space-Group and Crystal-Structure Determination. *Acta Crystallogr. Sect. A* **2015**, *71* (1), 3–8. https://doi.org/10.1107/S2053273314026370.

(5) Dolomanov, O. V; Bourhis, L. J.; Gildea, R. J.; Howard, J. A. K.; Puschmann, H. OLEX2: A Complete Structure Solution, Refinement and Analysis Program. *J. Appl. Crystallogr.* **2009**, *42* (2), 339–341. https://doi.org/10.1107/S0021889808042726.

(6) Sheldrick, G. M. Crystal Structure Refinement with {\it SHELXL}. *Acta Crystallogr. Sect. C* **2015**, *71* (1), 3–8. https://doi.org/10.1107/S2053229614024218.

(7) Spek, A. L. {\it PLATON} SQUEEZE: A Tool for the Calculation of the Disordered Solvent Contribution to the Calculated Structure Factors. *Acta Crystallogr. Sect. C* **2015**, *71* (1), 9–18. https://doi.org/10.1107/S2053229614024929.
